# Practical and Scalable Two-Step Process for 6-(2-Fluoro-4-nitrophenyl)-2-oxa-6-azaspiro[3.3]heptane: A Key Intermediate of the Potent Antibiotic Drug Candidate TBI-223

**DOI:** 10.1021/acs.oprd.3c00148

**Published:** 2023-07-12

**Authors:** Flavio S.P. Cardoso, Appasaheb L. Kadam, Ryan C. Nelson, John W. Tomlin, Dipendra Dahal, Christopher S. Kuehner, Gard Gudvangen, Anthony J. Arduengo, Justina M. Burns, Sarah L. Aleshire, David R. Snead, Fengrui Qu, Ken Belmore, Saeed Ahmad, Toolika Agrawal, Joshua D. Sieber, Kai Oliver Donsbach

**Affiliations:** † Medicines for All Institute, Virginia Commonwealth University, 737 N. 5th St., Box 980100, Richmond, Virginia 23298, United States; ‡ School of Chemistry and Biochemistry, Georgia Institute of Technology, Atlanta, Georgia 30332-0400, United States; § Department of Chemistry and Biochemistry, The University of Alabama, Tuscaloosa, Alabama 35487-0336, United States; ∥ Department of Chemistry, Virginia Commonwealth University, 1001 West Main Street, Richmond, Virginia 23284-3208, United States

**Keywords:** tuberculosis, TBI-223, azaspiro[3.3]heptane, spiroamine

## Abstract

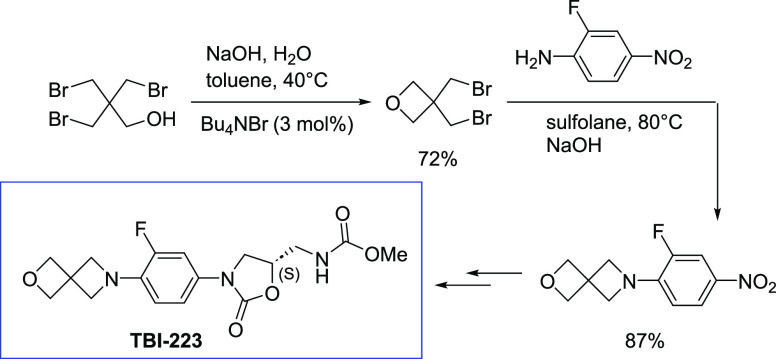

A low-cost, protecting group-free route to 6-(2-fluoro-4-nitrophenyl)-2-oxa-6-azaspiro[3.3]heptane (**1**), the starting material for the in-development tuberculosis treatment TBI-223, is described. The key bond forming step in this route is the creation of the azetidine ring through a hydroxide-facilitated alkylation of 2-fluoro-4-nitroaniline (**2**) with 3,3-bis(bromomethyl)oxetane (BBMO, **3**). After optimization, this ring formation reaction was demonstrated at 100 g scale with isolated yield of 87% and final product purity of >99%. The alkylating agent **3** was synthesized using an optimized procedure that starts from tribromoneopentyl alcohol (TBNPA, **4**), a commercially available flame retardant. Treatment of **4** with sodium hydroxide under Schotten–Baumann conditions closed the oxetane ring, and after distillation, **3** was recovered in 72% yield and >95% purity. This new approach to compound **1** avoids the previous drawbacks associated with the synthesis of 2-oxa-6-azaspiro[3,3]heptane (**5**), the major cost driver used in previous routes to TBI-223. The optimization and multigram scale-up results for this new route are reported herein.

## Introduction

Tuberculosis (TB) is a disease caused by an infection of *Mycobacterium tuberculosis* bacteria and is one of the leading causes of mortality worldwide, causing 1.5 million deaths in 2020 alone.^1−3^ TB is a preventable and curable disease. Unfortunately, most of the TB burden falls on low- and middle-income countries (LMICs), where the cost of treatment can be a significant burden for many households. In addition, the emergence of multidrug-resistant TB (MDR-TB) strains makes fighting this disease even more challenging. To combat this global health crisis, it is imperative that effective and inexpensive treatment options are available to everyone including those in the LMIC regions.

**Figure 1:**
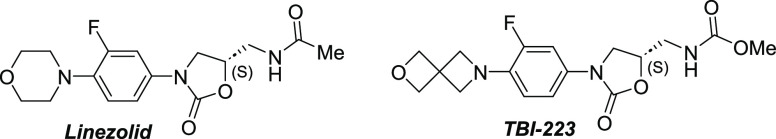
Current tuberculosis treatment linezolid and the in-development analog TBI-223.

Oxazolidinones are a well-known class of antibiotics used to treat *Mycobacterium* infections. Linezolid (Figure 1), an FDA-approved drug in this class of molecules, has shown activity against MDR-TB. Unfortunately, linezolid also exhibits toxicity toward blood and bone cells, which limits the overall treatment duration.^4^ TBI-223 is an in-development analog of linezolid, with early indications showing a similar positive therapeutic impact without the unwanted toxicity.^5,6^ However, continued clinical trials, eventual market uptake, and global affordability of this important drug candidate will be contingent on a low-cost, scalable route to the API.

**Scheme 1:**
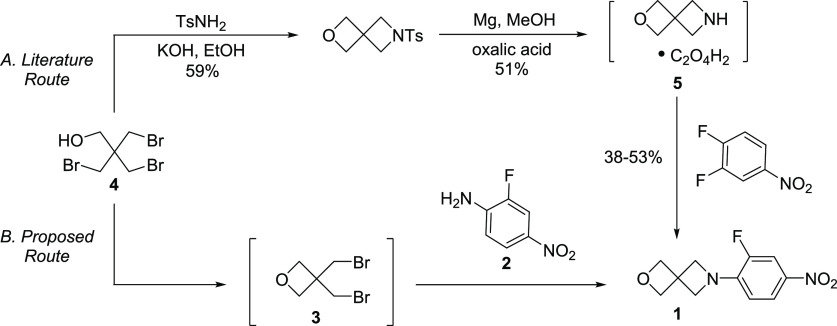
The Literature Route to Prepare Compound **1** and Our Proposed Alternative Route^7−10^

In 2017, the Global Alliance for TB Drug Development (TB Alliance) reported a synthesis of TBI-223^5^ based on preparative chemistry reported a year earlier,^7^ which passes through spirocyclic arylamine **1** (Scheme 1A). A technoeconomic analysis of this route identified intermediate **1** as the major cost driver for the API; hence, developing a less expensive process for its preparation would lead to significant cost reduction for future large-scale preparations of the API. The current route to **1** is depicted in Scheme 1 Route A and involves the initial preparation of the *oxa*-spiroazetidine **5** using a method based on an earlier report by Carreira et al.^8^ This route starts from tribromoneopentyl alcohol (TBNPA, **4**), a large-scale commercial flame retardant. Treatment of **4** with *p*-toluenesulfonamide simultaneously closes both the oxetane and *N*-tosyl protected azetidine rings. The subsequent reaction with magnesium turnings releases the free amine **5**. Unfortunately, this step is known to be inefficient on scale due to a sluggish filtration affecting the yield of the reaction.^9^ Isolation of the amine requires a final conversion to an oxalate salt that suffers from stability issues, precluding long-term storage of this intermediate.^9,10^ More recently, improved preparations of **5** were disclosed that use benzylamine for the azetidine ring formation^9,10^ and more thermally stable sulfonate salts for isolation.^10^ However, incorporating this chemistry into the TBI-223 route was problematic for two reasons. First, a Pd/C-catalyzed hydrogenation to remove the benzyl protecting could lead to some cost uncertainties because of the increasing price of palladium metal. Second, catalytic hydrogenation facilities may not be generally available to the broader global generics manufacturing market; outsourcing this hydrogenation step may lead to additional manufacturing costs that would impact the final price for the API in LMIC markets.

A more scalable route to **1** could be achieved by reordering the sequence of reactions (Scheme 1B), with the key step being a proposed double *N*-alkylation of 2-fluoro-4-nitroaniline with 3,3-bis(bromomethyl)oxetane (BBMO, **3**). Although anilines (especially with strongly electron-withdrawing aromatic substituents) are typically not considered good nucleophiles for alkyl halides, literature precedence for other azetidines being formed by the reaction between an aromatic amine and a bis(alkylhalide) was encouraging.^11^ The advantages of this approach are twofold. First, the oxetane can be formed using a known reaction from **4**(9,10) but without isolation of the problematic amine. Second, the necessary aniline can be prepared in a well-precedented, high-yielding S_N_Ar reaction between NH_3_ and 3,4-difluoronitrobenzene,^12−15^ the commodity starting material for linezolid. This report describes our work demonstrating this novel route.

## Results and Discussion

### Key Step: Proof of Concept—Alkylation of Aniline (**2**) with BBMO (**3**)

The investigation began with a screen of typical nitrogen alkylation conditions (Table 1) to validate the feasibility of the proposed key step: alkylation of **2** with **3**. At this early stage, the efficacy of these small-scale screening reactions was assessed by in-process control (IPC) HPLC area % of the crude reaction mixtures. Polar protic solvents such as water and ethanol (entries 1–3) gave little to no conversion to product, and utilization of polar, aprotic acetonitrile would require optimization beyond the scope of this current project (entries 4 and 5). In these cases, the crude reaction mixtures contained unreacted **2** with decomposition of BBMO (**3**).

**Table 1:**
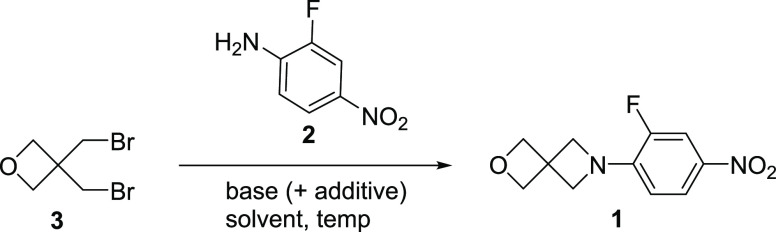
Aniline Alkylation Condition Screening^a^

entry	solvent	base	temp. (°C)	**1** (HPLC area % at 245 nm)^b^
1	EtOH	NaOH	reflux	3
2	EtOH	NaOEt	reflux	4
3	H_2_O	NaOH	80	
4	acetonitrile	K_2_CO_3_	reflux	6
5	acetonitrile	DBU	reflux	3
6	acetone	Cs_2_CO_3_	reflux	49
7	acetone	K_2_CO_3_^c^	reflux	6
8	acetone	K_2_CO_3_^d^	reflux	46
9	THF	NaH	20	40
10	DMF	NaH	20	44
11	DMF	NaOH	80	76
12	NMP	NaOH	80	68
13	diglyme	NaOH	80	82
14	diglyme	KOH	80	85
15	DMSO	NaOH	80	94
16	DMSO	KOH	80	93
17	sulfolane	NaOH	80	90
18	sulfolane	NaOH^e^	80	87

a General reaction conditions: aniline (**2**, 100 mg, 1 equiv), BBMO (**3**, 1.2 equiv), and base (2.5 equiv) were added to the solvent (2 mL) in a small vial. This mixture was heated to the listed temperature with stirring for 16 h.

b These HPLC data are from an IPC sample taken at the end of the reaction. The percentages are not adjusted for response factors.

c KI (0.1 equiv) was added to the reaction mixture.

d TBAI (0.1 equiv) was added to the reaction mixture.

e Delivered as 50 wt % NaOH (aq).

The first successful cyclization reaction was obtained in acetone (Table 1, entry 6), where a modest conversion to the product was observed. However, this success was predicated on the use of Cs_2_CO_3_, which is a prohibitively expensive reagent on scale. Because acetone refluxes at a considerably lower temperature (56 °C) than the earlier screening reactions (80 °C), it is possible that this reactivity is due to the higher solubility of this base. To test this hypothesis, two additional screening reactions were conducted in acetone with the less expensive base K_2_CO_3_. In the first reaction (entry 7), the reaction was run under Finkelstein conditions (0.1 equiv of KI) to generate an activated alkylating species. However, the conversion for this system was considerably lower than the Cs_2_CO_3_ system, suggesting that a stronger electrophile on its own is not sufficient for productive cyclization. In the second test reaction (entry 8), tetrabutylammonium iodide (TBAI, 0.1 equiv) was added as a phase transfer catalyst (PTC) to draw the inorganic base into solution. These conditions resulted in a conversion to **1** that was comparable to Cs_2_CO_3_. Unfortunately, to offset the additional costs and process mass intensity (PMI) associated with the usage of TBAI, this system would require considerable optimization to achieve high yields of the desired product while maintaining very low levels of PTC. However, these reactions did provide valuable insight into the reaction: specifically, the need for a soluble inorganic base suggests that deprotonation of aniline may be necessary for productive alkylation reactions.

This hypothesis was validated in the next batch of screening reactions using either strong bases to fully deprotonate the aniline or solvents that more effectively solubilize inorganic salts (Table 1, entries 9–16). Reactions with NaH (p*K*_a_[H_2_] = 35; p*K*_a_[*p*-nitroaniline] = 20.9)^16,17^ did show signs of modest conversion to product (entries 9 and 10). The results in these cases, however, were not compelling enough to justify the potential safety concerns, especially with the commonly used, but unsafe, combination of NaH/DMF.^18^ The weaker (and less expensive) base NaOH is reasonably soluble at elevated temperatures in dimethylformamide (DMF), *N*-methyl-2-pyrrolidone (NMP), diglyme, and DMSO, and these combinations proved to be the most efficient alkylation systems (entries 11–13), with nearly quantitative conversion in DMSO (94 area % of **1**). Switching to KOH did not significantly affect conversions (entries 14 and 16), which suggests that the nature of the cation plays a minimal role in these systems and again speaks to the hypothesis that deprotonation of the aniline is a key step in this process. Although the results with these systems were more promising, the potential for solvent decomposition under these conditions and the inherent risks this can entail^19,20^ were a concern for further scale-up. In an attempt to balance the efficacy of these systems with a safer solvent, sulfolane was explored as an alternative solvent.

The aniline alkylation reaction is similarly effective when conducted in sulfolane (Table 1, entry 17) as in its acyclic counterpart DMSO. This is perhaps not too surprising given the similar solvation properties of these two compounds; however, sulfolane is generally considered to be a safer solvent under these basic, higher-temperature reaction conditions.^21^ Sulfolane is manufactured on a large scale and is used in a variety of industrial applications,^22−24^ so the cost and availability of this solvent should still adhere to the goal of creating a scalable, low-cost route. However, both sulfolane and NaOH are typically used as mixtures with water: 3 and 50 wt %, respectively. To probe the effects of water on this system, a final alkylation reaction using 50 wt % NaOH in water (Table 1, entry 18) was shown to proceed with only a small reduction in conversion compared to its anhydrous counterpart. With validation of scalable alkylation conditions in hand, the next focus was the optimization of the reaction for scale-up.

### Key Step: Optimization of the Alkylation Reaction

**Figure 2:**
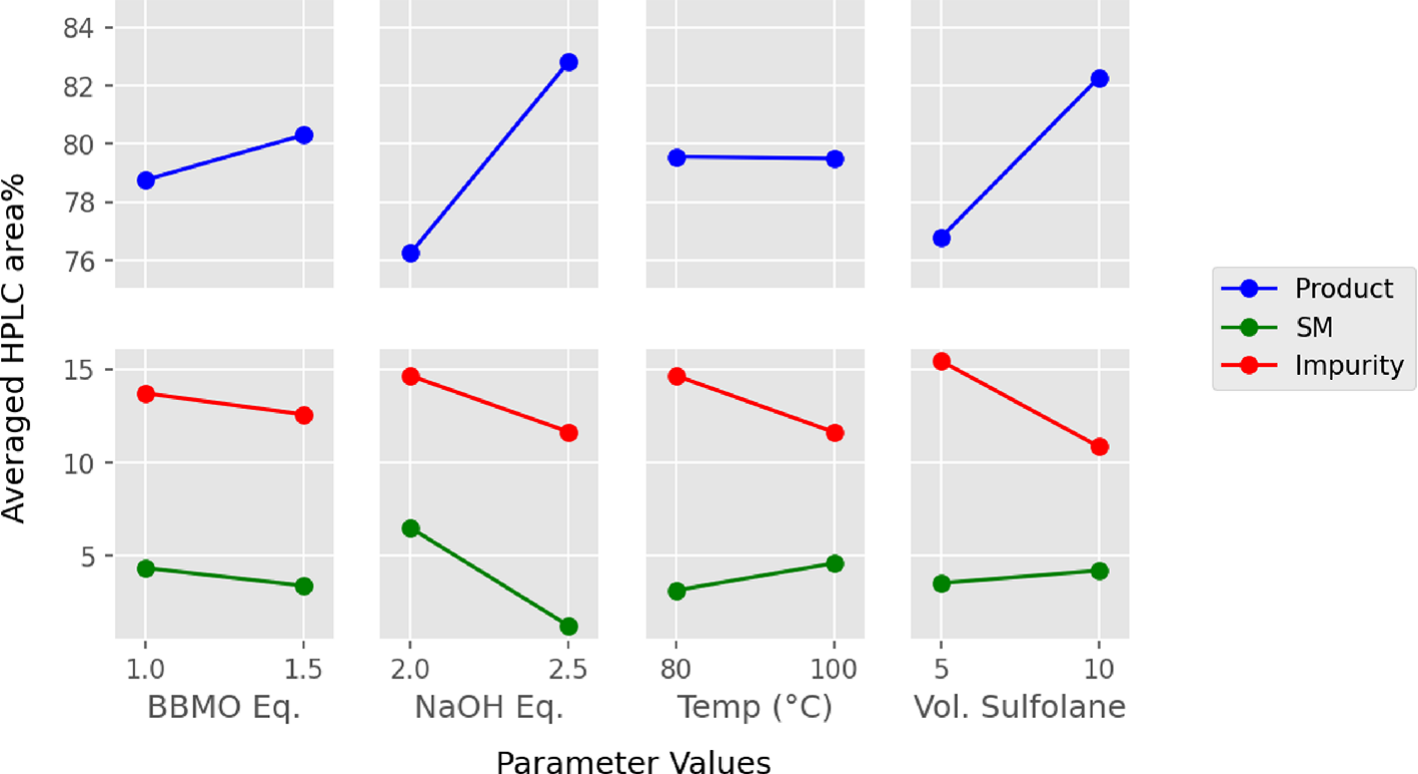
DOE mean plots for the two-level, full factorial DOE optimization of the main reaction parameters.

The optimization started with a two-level, full factorial DOE matrix constructed for four of the most important parameters in this system (2^4^ design):^25^ equivalents of **3** (1 and 1.5 equiv), equivalents of NaOH (2 and 2.5 equiv), temperature (80 and 100 °C), and volumes of sulfolane (5 and 10 V). In all experiments, the HPLC chromatograms were dominated by three species: product **1** and starting material **2**, which typically combined to ≥80 area %, and a single impurity, which contributed 10–15 area %. DOE mean plots^26^ for each parameter are shown in Figure 2. The complete data set and statistical analyses of these experimental data are provided in the Supporting Information. Statistically significant reductions in the impurity level were observed with increases in all four parameters (Figure 2, red lines), but increased equivalents of **3** had only a modest impact on formation of the side product (identified as compound **6**, *vide infra*). Conversions to **1** (Figure 2, blue lines), on the other hand, were only correlated to increases in either the equivalents of base or the volumes of solvent. On the basis of these results, the optimal conditions for scale-up would be slight excesses of NaOH and **3** and 10 V of solvent. Although increased temperatures did result in a modest reduction in the impurity, the lack of improvement in product formation tends to favors the lower reaction temperature (80 °C) for scale-up.

**Figure 3:**
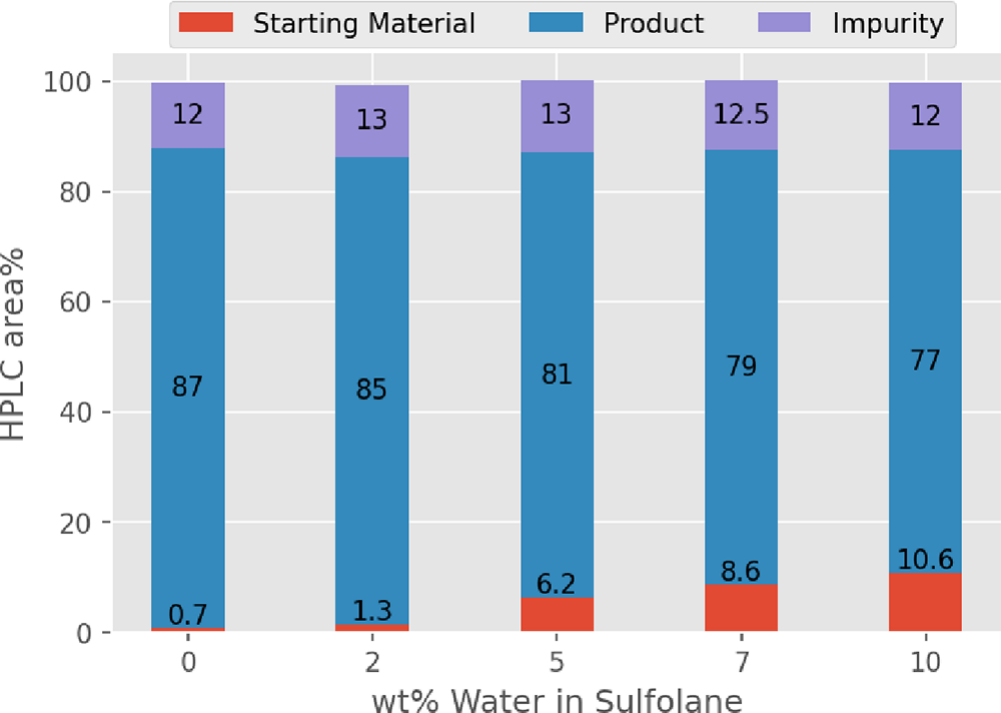
Effect of water on alkylation conversion and the ratio of alkylation products.

Although earlier validation reactions suggested a mild dependence on the presence of water on the alkylation conversion, this relationship was further explored to understand if there was a point of failure associated with adventitious water. To accomplish this, a series of reactions were conducted with increasing amounts of water, as expressed in wt % in sulfolane (Figure 3). With increasing amounts of water, there is a clear decrease in the consumption of **2** at the expense of **1**. A likely culprit for this trend is the decomposition of the alkyl bromide **3**. This hypothesis could not be fully confirmed as **3** is not detected in the HPLC analysis. Interestingly, the amount of impurity was nearly unchanged at all concentrations of water. Given the relatively high levels of this impurity (between 10–15 area %), the identification and minimization of this unwanted side product became the next focus.

### Key Step: Impurity Characterization and Inhibition of Formation

**Scheme 2:**
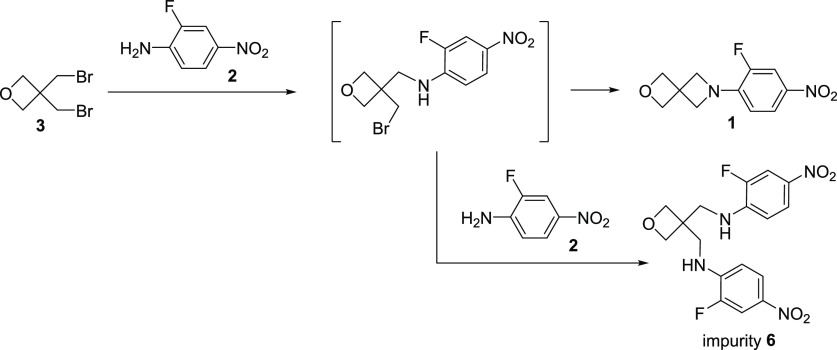
Potential Mode of Formation for Bis-aniline Impurity **6**

To characterize the reaction impurity, an analytical sample was isolated from a batch of crude reaction product (∼9:1 **1** to impurity as determined by HPLC). Trituration of the crude product with MTBE provided a sufficiently pure sample of the side product for characterization purposes. The NMR and MS data for the impurity suggest a bis-aniline adduct of BBMO (**6**, Scheme 2). This characterization is also consistent with the DOE observation that impurity reduction is correlated to the additional solvent. Specifically, the rate of the undesired second-order intermolecular alkylation should be more impacted by concentration than the rate of the first-order intramolecular ring closure. Unfortunately, additional increases in solvent volume to further reduce the amount of **6** would be detrimental to the overall reaction throughput and cost, and additional operational changes for improving the reaction outcome were explored.

To suppress the formation of **6**, a series of operational changes to the mode of addition were explored, as detailed in Table 2. As expected, a control reaction where all reagents were added together before heating (entry 1), which is equivalent to the earlier screening reaction conditions, resulted in a roughly 8:1:1 ratio of **1**, **2**, and **6**. Given the putative structure of **6**, the concentrations of the nucleophile and base must play the largest role in dictating the rate of impurity formation, so a series of slow reagent additions to hot mixtures of the remaining reagents were tested, with the hope that this slow addition would limit the instantaneous concentration of these species. Addition of the nucleophile **2** as a solution in sulfolane to the hot reaction mixture (entry 2) did result in a small decrease in the level of impurity; however, a concomitant decrease in **1** formation was also observed. This is consistent with the earlier hypothesis that **3** can slowly decompose in the presence of basic water, and by limiting the amount of nucleophile, this decomposition pathway becomes more competitive with the productive alkylation reactions. A major process improvement came when NaOH (as a 50 wt % solution in water) was slowly added to the hot reaction mixture (entry 3). In this case, near complete consumption of **2** was observed with a mirrored increase in **1**. Because the base deprotonates **2** at a much faster rate than it decomposes **3** (as inferred by comparison of the amounts of **1** and unreacted **2** in the control reactions), slow addition appears to limit the amount of base that is available to decompose **3**. As expected based on the slow-addition hypothesis, dropwise addition of the electrophile **3** to the hot reaction mixture did not affect the reaction outcome (entry 4). Although these initial slow addition tests had only a limited effect on impurity levels, these experiments did provide some valuable insights into further process improvements.

**Table 2:** Effect of Mode of Addition on the Outcome of the Alkylation Reaction^a^

entry	mode of addition	HPLC IPC (area % at 245 nm)^b^		
		SM	product	impurity
1	all reagents added at room temp. and heated to 80 °C	10	79	11
2	dropwise addition of a solution of aniline in sulfolane to the hot reaction mixture	13	78	9
3	dropwise addition of 50 wt % NaOH (aq) to the hot reaction mixture	3	86	8
4	dropwise addition of a solution of bromo-oxetane in sulfolane to the hot reaction mixture	10	77	12
5	separate dropwise addition of both aq NaOH and aniline solution to the hot reaction mixture	3	90	5
6	dropwise addition of a solution of aniline and bromo-oxetane in sulfolane to the hot reaction mixture	4	92	2

a General reaction conditions: aniline (**2**, 1.0 g, 1 equiv), BBMO (**3**, 1.2 equiv), NaOH (2.5 equiv), and sulfolane (10 mL) were added together as described in the table. The temperature was 80 °C for all reaction, and the overall reaction time was 3 h. In the cases where two sulfolane solutions were added together, half of the solvent was used to make each individual solution so that the final reaction volume would remain fixed.

b These percentages are not adjusted for response factors.

Because the desired alkylation reaction competes with two undesired reaction paths, base decomposition of **3** and formation of **6**, it is reasonable that a multicomponent slow addition may provide the reaction control needed to achieve high conversions to **1** with minimal competition from side reactions. Indeed, improved conversion to **1***and* reduced levels of **6** were finally achieved by slow addition of separate solutions of **2** and NaOH (50 wt % in water) (Table 2, entry 5). Although this new process provided far superior results, slow addition of two separate solutions to the reaction mixture was deemed to be a bit cumbersome for scale-up. Minimizing the concentrations of **3** and **2** during the reaction would also slow the rates of decomposition and secondary alkylation reactions. Thus, in a final modification, a mixed solution of **3** and **2** was slowly added to a hot mixture of NaOH in sulfolane. This mode of addition proved to be the most successful reaction system, with product levels in excess of 90 area % and starting material and impurity levels both below 5 area % (Table 2, entry 6). This method benefits from maintaining a low concentration of **2**, wherein cyclization (a first-order kinetic process) is favored over impurity **6** formation (a second-order kinetic process).

From a practical standpoint, this final operational method (Table 2, entry 6) provides several additional benefits. The first benefit is that adding a premixed solution of **3** and **2** reduces the operational complexity of the system. The second, perhaps less obvious, benefit is related to both the practical usage of sulfolane and the total amount of water in the reaction mixture. Pure sulfolane is a solid at room temperature. To make it easier to transfer, industrial-grade sulfolane typically contains 3 wt % water, which has a melting point below room temperature. Solid NaOH is soluble in this solvent mixture, so premixing these components before reactant addition ensures that the overall water content stays at a minimum, controlled level. With this complete set of reaction and process optimizations in hand, the next goal was to demonstrate product isolation from the final reaction mixture.

### Key Step: Product Isolation

Although the optimized reaction conditions appear to give clean conversion to **1**, isolation from the reaction mixture remained a significant challenge due to the high boiling point of sulfolane. Sulfolane and water are miscible, so addition of sufficient water to the reaction mixture should cause the hydrophobic product to precipitate as an easily isolable solid. Indeed, addition of water to the room temperature reaction mixture causes precipitation of **1** as a light-yellow to light-green solid. However, reproducible product purities were not achieved by this method, and large amounts of water were necessary for full recovery (40 V), which raised a critical throughput concern. Attempts to use less water by lowering the reaction mass temperature were unsuccessful. Because a slight excess of base was used in the alkylation reaction, it was postulated that pH may be a crucial factor in the isolation. Neutralization of the crude reaction mixture with 1 N HCl proved to be the key for successful product isolation, a process that also generates NaCl that may affect product solubility. After neutralization, reproducible recovery of **1** was achieved after addition of only 10 V of water, with isolated yields between 80 and 85% and purities of >95 wt %.

### X-ray Crystallographic Characterization of **1**

**Figure 4:**
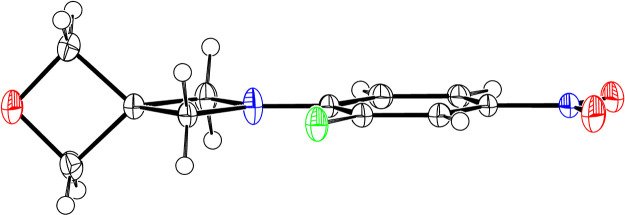
ORTEP depiction (50% ellipsoids) of **1**. Disorder in the fluorine position has been omitted for clarity.

To produce pure material for the HPLC wt % assay, **1** was recrystallized by slow cooling of a hot solution of **1** in ethanol. This process not only improved the purity of the product, but it also produced highly crystalline, clear yellow rods. The availability of high-quality crystals of **1** enabled a detailed structural analysis by single crystal X-ray diffraction studies (Figure 4). Taken together with ^14^N solution NMR data, the solid-state structure provides insights into the geometry and flexibility of this particular spiro-azetidine moiety. These detailed structural features might be reflected in the reactivity of this pharmaceutical intermediate and/or its active site binding ability as a pharmacophore.

Azetidines are recognized to have inversion barriers higher than acyclic amines, but these barriers are substantially influenced by substituents, with electron accepting *N*-aryl moieties markedly lowering the barriers to inversion.^27^ Nonetheless, many structures in the Cambridge Crystallographic Database show some nonplanarity in *N*-arylazetidines and a degree of pyramidalization at the azetidine nitrogen.^8,28,29^ In solution, the ^14^N magnetic resonance for the azetidine nitrogen in **1** was observed at −312.29 ppm with a line width of 1470 Hz. The quadrapolar relaxation of the ^14^N nucleus normally precludes the observation of amines in all but the smallest and most symmetric molecular environments.^30−32^ The observation of the ^14^N resonance in **1** suggests that the nitrogen is close to planar (or at least is rapidly inverting through a low barrier) in a pseudo-C_2v_ environment. Spiroazetidine **1** is relatively large and fairly asymmetric (considering the fluoronitroaromatic group), and these features contribute to a broadening of even the ^14^N resonance in the nitro-group (δ −8.12, 285 Hz) compared to the 110 Hz line width in nitrobenzene.^33^

In the solid-state structure, molecules of **1** reside in a crystallographically imposed mirror plane so that the azetidine moiety is completely planar. The fluorine adjacent to the spiroazetidine moiety is disordered between the two “*ortho*-positions”. There is a slight elongation of the azetidine-nitrogen’s thermal ellipsoid in the direction perpendicular to the crystallographic mirror plane that is not reflected in the (more spherical) thermal ellipsoid of the nitrogen center of the nitro-group. This out-of-plane thermal motion for the azetidine can be interpreted as a very weak tendency of the azetidine toward pyramidalization. However, the overall quality of the structure and the compactness of the ellipsoids suggest that it is most appropriate to regard the molecule as essentially planar in the solid state and in solution (as supported by the ^14^N NMR spectroscopy).

By way of comparison, most spiroazetidine structures currently found in the crystallographic literature bear an aliphatic carbon as the substituent exocyclic to the azetidine. For these latter structures, the azetidine nitrogen rises an average of 530 pm above the place of the three attached carbons. 4-(2-Oxa-6-azaspiro[3.3]hept-6-yl)benzonitrile is the single spiroazetidine appearing in the CCDC database (NUQSEY) that bears an aryl substituent (*p*-benzonitrile) at the azetidine nitrogen. The tendency of the aryl moiety to reduce the barrier to inversion at nitrogen in this benzonitrile derivative is further enhanced by the electron-withdrawing nitrile group. Nonetheless, the azetidine nitrogen in 2-oxa-6-azaspiro[3.3]hept-6-yl)benzonitrile rises only 212 pm out of the plane of the three attached carbons.

Because of the electronic character of the spiroazetidine substituent (an *N*-oxazolidinone) in TBI-223, the inversion barrier at the spiroazetidine can be expected to be higher than that found in **1**.

### BBMO (**3**) Synthesis and Scale-up

**Scheme 3:**
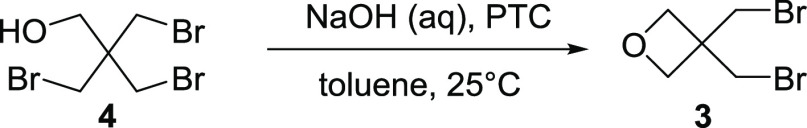
Synthesis of **3** from **4**

Although **3** is commercially available in lab-scale quantities, the limited availability on larger scales was a concern for ultimately creating a low-cost route, so to this end, the focus shifted to ensuring that a scalable synthesis of **3** was possible. Oxetane syntheses are well preceded in the literature and are typically accomplished via base-driven 3-halopropanol cyclization reactions.^8,9,34^ For example, the previous syntheses of amine **5** start from the alkyl bromide **4**, a commercial flame retardant that is more accessible on larger scales, but isolation of the amine from these systems proved problematic. However, one of these synthetic routes, as described by AstraZeneca, claimed to pass through **3** as a reactive intermediate.^9^ In this work, the oxetane ring closure was accomplished using toluene/NaOH (aq) Schotten–Baumann conditions, with the PTC tetrabutylammonium hydrogen sulfate (TBAHS) catalyzing the reaction (Scheme 3), and the toluene layer containing crude **3** was used directly in the subsequent reaction. The scalability and low cost of these conditions were attractive; thus, studies as to whether these solutions of **3** could also be employed under the alkylation reaction conditions described above were undertaken. The reported oxetane synthesis conditions (0.05 equiv TBAHS, 2.0 equiv NaOH) worked reasonably well, with crude **3** being isolated as a toluene solution in 80–85% assay yields. Unfortunately, addition of **2** to the reaction mixture directly or attempts to use the crude toluene mixtures of **3** in the sulfolane/NaOH reaction conditions were unsuccessful, resulting in lower yields and purities of the desired spiroamine product. This is perhaps unsurprising as there may be a variety of alkylating species present in these mixtures that react with aniline **2**, leading to a variety of unwanted and difficult to remove alkylation products. Isolation of **3** from the toluene solution was possible by vacuum distillation, but the isolated purities using this synthetic route were low owing to the presence of these byproduct impurities. To increase the quality of the isolated BBMO, optimization of these synthetic conditions was necessary.

To retain the benefits of the low-cost Schotten–Baumann conditions, optimization efforts focused on the PTC and the base. Although many potential PTCs could catalyze this reaction, precedence for improved yields of **3** using tetrabutylammonium bromide (TBABr) as the PTC^35,36^ suggested that this catalyst may lead to a cleaner reaction. In addition, the near stoichiometric amount of base in the earlier procedure may ultimately limit the productive conversion to product, so the amount of NaOH was increased. The modifications proved successful; with TBABr (3 mol %) and an excess of base (3.5 equiv), crude **3** was obtained in 83% assay yield at a 65 g scale. A single major impurity, 3-bromo-2-(bromomethyl)-1-propene, was identified in these crude samples by comparison with a commercially available sample. This side product arises from a double elimination of formaldehyde and bromide after the initial alcohol deprotonation.^37^ Vacuum distillation of the crude product largely removes this impurity, and the isolated yield and purity after distillation were 72% at >95% wt %, respectively, which are the same quality as the commercial sources used for the alkylation screening reactions. With both steps of the proposed route validated and optimized, the efforts turned to testing the newly developed two-step procedure at a reasonable scale.

### Scaled Demonstrations of the Full Process

**Scheme 4:**
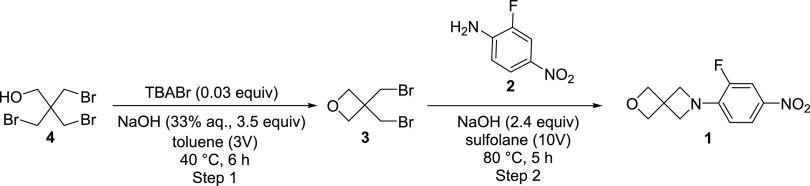
Two-Step Synthesis of **1** from TBNPA (**4**) and 2-Fluoro-4-nitroaniline (**2**)

Both steps in the optimized procedure (Scheme 4) were conducted several times at a variety of scales, and these experiments demonstrated a surprisingly robust process. The synthesis of **3** was reproduced on scales up to 400 g (Table 3). Although the IPC data suggest a slight decrease in the amount of oxetane as the scale increases,^38^ the isolated yields (72% average) and purities (96.6 wt % average) were almost identical for each run. The final alkylation reaction was also demonstrated on a variety of scales (Table 4) using our synthetic **3** rather than commercial sources. Again, the IPC data show a very similar run-to-run reaction profile, and after precipitation of the final product from the crude reaction mixture, **1** was isolated in excellent yields in all runs (85.5% average). A marked improvement in product purity was observed with increasing reaction scale (up to 99 wt % at 100 g), which suggests that isolation from the crude reaction mixture becomes more efficient at larger scales.

**Table 3:** Large-Scale Syntheses of **3** under Optimized Conditions (Step 1, Scheme 4)

entry	scale (g)	GCMC IPC (TIC area %)	crude product		purified product (after distillation)			
			wt (g)	purity (wt %)	wt (g)	GCMS (TIC area %)	purity (wt %)	yield (%)
1	65	87	46.5	86.8	37	95.9	95.4	72
2	200	85	153	86.0	110	99.2	98.3	72
3	400	82	295	85.0	220	99.7	96.0	72

**Table 4:** Large-Scale Syntheses of **1** under Optimized Conditions (Step 2, Scheme 4)

entry	scale (g)	HPLC IPC (area % at 245 nm)	isolated product			
			wt (g)	HPLC (area % at 245 nm)	purity (wt %)	yield (%)
1	5	92.7	7.1	96.2	95.0	84
2	10	91.2	13.6	97.6	91.0	84
3	50	92.6	66.4	99.5	99.3	86
4	100	94.0	135	100	99.0	88

## Conclusions

By re-evaluating the order of the previous bond forming reactions, an improved method to prepare 6-(2-fluoro-4-nitrophenyl)-2-oxa-6-azaspiro[3.3]heptane (**1**), a key intermediate and cost driver for the in-development TB treatment TBI-223, has been developed. This approach is more efficient than the previous methods utilizing **5**, a high-cost building block, because it avoids a tedious tosylamine deprotection step and it bypasses the synthesis and isolation of an unstable oxalate salt. In addition, an improved synthetic method was developed for the alkylating agent **3**, which is an important synthon for a number of dialkyloxetane systems. This improved process was demonstrated at 100 g scale in an overall yield of 62%, which is more than double the yield of the previous route to **5**.^39^ These improvements will ultimately provide a more accessible, lower-cost alternative to this important API intermediate.

## Experimental Section

All materials were purchased from Combi-Blocks, Ambeed, Aldrich Chemical Company, Fisher Scientific, Alfa Aesar, Acros Organics, or TCI. Solid reagents were used without further purification. For all compounds, ^1^H and ^13^C NMR spectra were recorded on a Bruker Avance III 400, 600, or 700 MHz spectrometer. Chemical shifts were measured relative to the solvent resonance for ^1^H and ^13^C NMR (CDCl_3_ = 7.26 ppm for ^1^H and 77.0 ppm for ^13^C, DMSO-*d*_6_ = 2.50 ppm for ^1^H and 39.5 ppm for ^13^C, CD_3_OD = 3.31 ppm for ^1^H and 49.0 ppm for ^13^C, D_2_O = 4.79 ppm for ^1^H) and relative to internal standards for ^14^N NMR (CH_3_NO_2_) and ^19^F NMR (CFCl_3_). Coupling constants (J) are reported in hertz (Hz). The following abbreviations were used to designate signal multiplicity: s, singlet; d, doublet; t, triplet; q, quartet; p, pentet; dd, doublet of doublets; ddd, doublet of doublet of doublets; dt, double of triplets; ddt, doublet of doublet of triplets; m, multiplet; br, broad. Reactions were monitored by HPLC or GCMS using the methods described in the Supporting Information. Glassware was oven-dried at 120 °C, assembled while hot, and cooled to ambient temperature under an inert atmosphere. Unless noted otherwise, reactions were performed under a nitrogen atmosphere. A full hazard assessment and differential scanning calorimetry are recommended before further scale-up of this process.

### Preparation of 3,3-Bis(bromomethyl)oxetane (**3**)

To a 2000 mL three-neck round-bottom flask fitted with an overhead stirrer equipped with a PTFE paddle, toluene (1200 mL), tribromo alcohol **4** (400 g, 1.23 mol, 1 equiv), and tetrabutylammonium bromide (11.9 g, 0.036 mol, 0.03 equiv) were added at room temperature, at which point the reaction mixture was warmed to 40 °C (internal temperature). A separately prepared solution of 8.2 M aqueous NaOH (525 mL, 3.5 equiv) was added dropwise within 15 min while maintaining 40 °C. The reaction progress was monitored by GCMS. After completion (typically 6 h), the reaction mixture was cooled to room temperature, and the water layer was removed. The toluene layer was washed with distilled water (4 × 250 mL) until the pH of the water washes became neutral. The organic layer was concentrated under a vacuum to obtain 295 g of crude product **2** (85% GCMS TIC area % purity).

Crude BBMO was purified by short path vacuum distillation (a detailed assessment of the distillation is provided in the Supporting Information). Low-boiling fractions collected at vapor temperatures between 70 and 85 °C were discarded. The major fraction (220 g) was collected at a vapor temperature of 85 °C. This fraction was found to be a nearly pure product (99.7% GCMS area % purity; 96% wt % purity), and the yield of this fraction based on purity was 72%. ^1^H NMR (600 MHz, CDCl_3_) δ 4.43 (s, 4H), 3.85 (s, 4H); ^13^C NMR (151 MHz, CDCl_3_) δ 77.8 (2C), 44.9, 36.9 (2C). The ^1^H NMR spectroscopic data match previously reported values.^10^

### Preparation of 6-(2-Fluoro-4-nitrophenyl)-2-oxa-6-azaspiro[3.3]heptane (**1**)

To a 2000 mL three-neck round-bottom flask fitted with an overhead stirrer equipped with a PTFE paddle, sulfolane (600 mL, containing 3% water) followed by solid NaOH (61.5 g, 1.53 mol, 2.4 equiv) was added at room temperature. The reaction mixture was heated to 80 °C (internal temperature) and stirred until NaOH dissolved completely (approximately 15 min). To this, a solution of aniline **2** (100 g, 0.64 mol, 1 equiv) and BBMO **3** (195.3 g, 0.768 mol, 1.2 equiv, 96 wt % purity) dissolved in sulfolane (400 mL, 3 wt % water) was added dropwise using a syringe pump at 80 °C over a period of 2 h. The reaction mixture was maintained at this temperature with stirring until the reaction was complete as determined by HPLC (approximately 3 h, see Figure S6 for a representative HPLC chromatogram).

Water (500 mL, room temperature) was added to the reaction mixture at 80 °C. The reaction mixture was then allowed to cool to 30 °C. A solution of HCl (1 N, 210 mL) was added dropwise with stirring while maintaining the internal temperature at 25–30 °C to adjust the pH to between 5 and 6 (during the addition of 1 N HCl, a color change was observed from dark yellow to green). Excess water was then added to the reaction mixture to adjust the total volume of water and 1 N HCl to 10 V. The slurry was stirred for an additional 10 min. The precipitated product was filtered through a Buckner funnel and washed with water (2.5 V × 8 = 20 V). The isolated lime-green solid was dried under a vacuum at 50 °C for 24 h to obtain 135 g of product (100% HPLC area % purity; 99% wt % purity, 87% yield based on purity). A representative HPLC chromatogram of the pure product is shown in Figure S7 (Supporting Information). ^1^H NMR (600 MHz, DMSO-*d*_6_) δ 7.94–7.91 (m, 2H), 6.55 (t, *J* = 9.4 Hz, 1H), 4.72 (s, 4H), 4.35 (s, 4H); ^13^C NMR (151 MHz, DMSO-*d*_6_) δ 148.88 (d, *J* = 242.7 Hz), 143.86 (d, *J* = 10.7 Hz), 136.04 (d, *J* = 7.7 Hz), 121.90 (d, *J* = 1.5 Hz), 112.37 (d, *J* = 5.5 Hz), 111.76 (d, *J* = 22.9 Hz), 79.58 (s), 62.20 (s), 39.05 (d, *J* = 2.6 Hz). These spectroscopic data match previously reported values.^7^

A sample of crude **1** was further purified by crystallization from warm ethanol. The recrystallized sample exhibited a melting point of 204–206 °C. ^1^H NMR (700 MHz, CDCl_3_) δ 7.94 (ddd, 1H), 7.86 (ddd, 1H), 6.31 (apparent t, {dd = 9.4 Hz, 1H}), 4.86 (s, C*H*_2_O, 4H), 4.35 (s, C*H*_2_N, 4H); ^19^F NMR (658.8 MHz, CDCl_3_, ref. CFCl_3_) δ −133.92; ^13^C NMR (176 MHz, CDCl_3_) δ 149.68 (d, *C*F, *J* = 243.5 Hz), 143.37 (d, FC*C*N, *J* = 10.6 Hz), 137.72 (d, *C*NO_2_, *J* = 7.6 Hz), 121.60 (d, FCC*C*H, *J* = 1.5 Hz), 112.22 (d, H*C*CF, *J* = 5.6 Hz), 111.60 (d, FCCC*C*H, *J* = 22.8 Hz), 80.76 (s, *C*H_2_O), 62.63 (s, *C*H_2_N), 39.71 (d, *C*_4_*C J* = 2.4 Hz). ^14^N NMR (36.14 MHz, CDCl_3_, ref. CH_3_NO_2_) δ −8.12 (width = 286), −312.3 (width = 1470 Hz). UV–vis (CH_2_Cl_2_) λ_max_ 384 nm (ε 1941 M^–1^ cm^–1^). Anal. calc. for C_11_H_11_N_2_O_3_: C, 55.46; H, 4.65; N, 11.76; found: C, 54.87; H, 4.49; N, 11.63.

## Supporting Information Available

Additional experimental details, analytical methods, and details of the X-ray structure determination on **1** (PDF)
