# Supplementary material for: Practical and Scalable Two-Step Process for 6-(2-Fluoro-4-nitrophenyl)-2-oxa-6-azaspiro[3.3]heptane: A Key Intermediate of the Potent Antibiotic Drug Candidate TBI-223
Source: Org Process Res Dev. 2023 Jul 12;27(7):1390–9. doi: 10.1021/acs.oprd.3c00148 (PMC10367134; doi:10.1021/acs.oprd.3c00148)
Supplement: Supplementary file 1 — op3c00148_si_001.pdf [file op3c00148_si_001.pdf]

Supporting Information

Practical and scalable two-step process for 6-(2-fluoro-4-nitrophenyl)-2-oxa-6-azaspiro[3.3]heptane: A key intermediate of the potent antibiotic drug candidate TBI-223

Flavio S. P. Cardoso,[a] Appasaheb L. Kadam,[a] Ryan C. Nelson,[a] John W. Tomlin,[a] Dipendra Dahal,[b] Christopher S. Kuehner,[b] Gard H. Gudvangen,[b] Anthony J. Arduengo III,[b] Justina M. Burns,[a] Sarah L. Aleshire,[a] David R. Snead,[a] Fengrui Qu,[c] Ken Belmore,[c] Saeed Ahmad,[a] Toolika Agrawal,[d] Joshua D. Sieber[d] , Kai Donsbach*[a]*

[a] *Medicines for All Institute, 737 N. 5th St., Box 980100, Richmond VA, 23298 U.S.A.*

[b] *School of Chemistry and Biochemistry, Georgia Institute of Technology, Atlanta, GA, 30332-0400, U.S.A.*

[c] *Department of Chemistry and Biochemistry, The University of Alabama, Tuscaloosa, AL 35487-0336, U.S.A.*

[d] *Department of Chemistry, Virginia Commonwealth University, 1001 West Main Street, Richmond, VA 23284-3208.*

Email: m4all@vcu.edu

Contents

Final Optimized Procedure 3

Step 1: Purification of Crude 3,3-Bis(bromomethyl)oxetane (3) 3

Step 2: Optimization, Impurity, and Additional Reaction Information 5

DOE Optimization of Alkylation Conditions: Data and Analysis Information 5

Isolation of an Analytical Sample of Step 2 Impurity (**6**) 8

HPLC Data of Reaction Mixture and Product 10

Photos of the Reaction and Isolated Product 11

References..12

NMR Spectra 13

GCMS Method Report..18

HPLC-UV Method Report...21

X-Ray Crystallographic Data and Analysis...24

Final Optimized Procedure

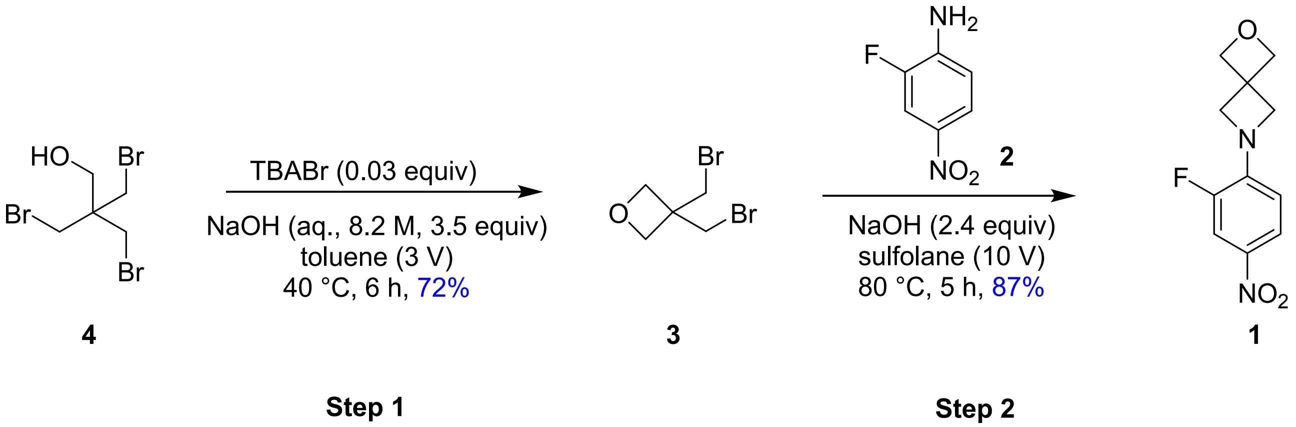

Step 1: Purification of Crude 3,3-Bis(bromomethyl)oxetane (3)

The crude sample of BBMO (295 g) was purified via short path distillation at 10 mbar pressure. The three fractions were collected, with their composition shown in Table S1 based on the GCMS data shown in Figure S1. The major impurity, 3-bromo-2-bromomethyl-1-propene (RT 2.4 min, matched to a commercial sample), is separated from the rest of the mixture in the initial stage of the distillation. The third major fraction was pure product (>96% wt% purity by GC). Yield of the pure fraction based on purity was 72% (99.7% GC A% purity).

Fraction No.	Vapor Temperature (°C)	Wt. (g)	Purity after distillation	
			GCMS TIC area%	wt%
1	<70	22	44.9	39.4
2	70-85	11	78.0	65.3
3	85	220	99.7	96.0

Table S1: Details of fraction collected during distillation.

AK-TBI-070-F1

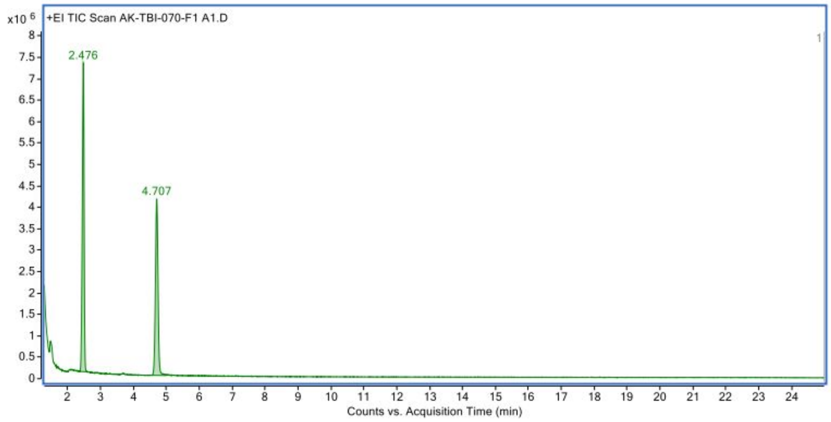

AK-TBI-070-F2

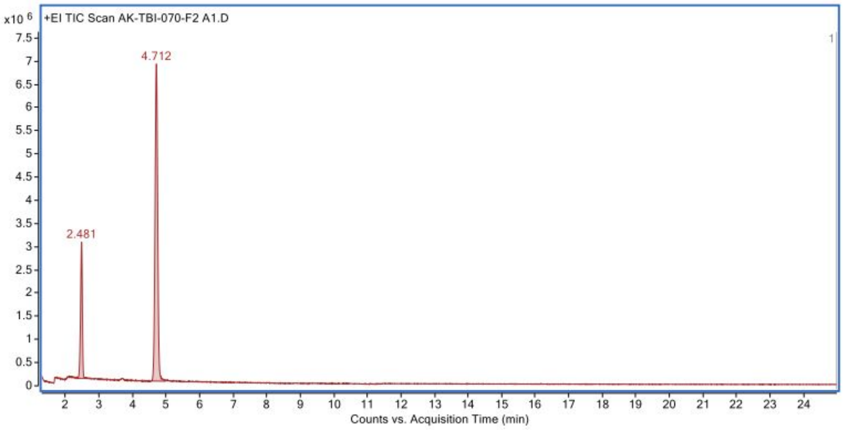

AK-TBI-070-F3

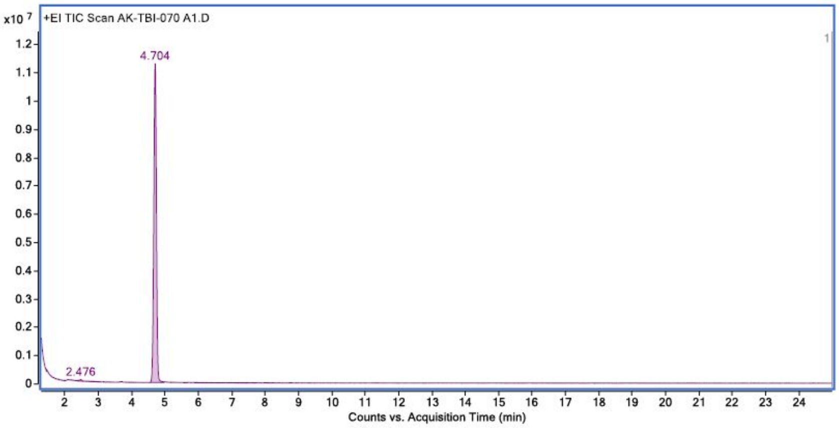

Figure S1: GCMS chromatograms of the different fractions collected during the distillation of crude BBMO (for chromatography conditions, see below).

Step 2: Optimization, Impurity, and Additional Reaction Information

DOE Optimization of Alkylation Conditions: Data and Analysis Information

Table S2. Reaction screening results for a DOE optimization of the alkylation of **2** with **3**.[1]

					HPLC area% at 245nm[2]		
Entry	Eq. **3**	Eq. NaOH	Temp (°C)	Solvent (V)	**3**	**1**	**6**
1	1	2	80	5	5.3	74.0	19.5
2	1	2	80	10	8.2	77.0	13.8
3	1	2	100	5	6.6	73.3	16.3
4	1	2	100	10	9.3	76.9	11.1
5	1	2.5	80	5	1.1	77.2	17.4
6	1	2.5	80	10	0.6	86.7	11.5
7	1	2.5	100	5	2.1	79.0	12.5
8	1	2.5	100	10	1.6	86.0	7.6
9	1.5	2	80	5	4.5	72.3	18.4
10	1.5	2	80	10	3.4	82.3	12.4
11	1.5	2	100	5	5.4	76.8	14.4
12	1.5	2	100	10	9.3	77.4	11.3
13	1.5	2.5	80	5	1.8	80.5	14.2
14	1.5	2.5	80	10	0.3	86.5	10.1
15	1.5	2.5	100	5	1.5	81.2	10.9
16	1.5	2.5	100	10	1.0	85.4	9.0

[1] General reaction conditions: NaOH was added to a solution of aniline **2** (100 mg, 1.0 eq.) and **3** in sulfolane. This mixture was then heated to the specified temperature for 3 h. [2] These are HPLC area% data for IPC samples taken of the reaction mixture. The area% are not corrected for the response factors of each compound.

These DOE data were first coded and then analyzed using ordinary least squares (OLS) methods as implemented by Statsmodels,[1] a statistics packs for the Python programming language. Initially,

the data were fit with a full model consisting of all the main effects and their interaction terms. However, the interactions terms were not found to be statistically insignificant (p value > 0.05), so they were ignored. The OLS results for the main effect term models are shown found in Table S3 for **1** and Table S4 for **6**.

Table S3: Ordinary least squares fit results for the level of **1** as indicated by the IPC data.

	**OLS Regression Results**					
**Dep. Variable:**	Product	**R-squared:**	0.888			
**Model:**	OLS	**Adj. R-squared:**	0.847			
**Method:**	Least Squares	**F-statistic:**	21.75			
		**Prob (F-statistic):**	3.51E-05			
**Time:**	7:35:39	**Log-Likelihood:**	-29.666			
**No. Observations:**	16	**AIC:**	69.33			
**Df Residuals:**	11	**BIC:**	73.2			
**Df Model:**	4					
**Covariance Type:**	nonrobust					
	**coef**	**std err**	**t**	**P>|t|**	**[0.025**	**0.975]**
**Intercept**	79.5313	0.466	170.696	0	78.506	80.557
**BBMO**	0.7687	0.466	1.65	0.127	-0.257	1.794
**NaOH**	3.2812	0.466	7.042	0	2.256	4.307
**Temp**	-0.0313	0.466	-0.067	0.948	-1.057	0.994
**Solvent**	2.7438	0.466	5.889	0	1.718	3.769
**Omnibus:**	1.493	**Durbin-Watson:**	2.553			
**Prob(Omnibus):**	0.474	**Jarque-Bera (JB):**	0.878			
**Skew:**	0.132	**Prob(JB):**	0.645			
**Kurtosis:**	1.883	**Cond. No.**	1			

Table S4: Ordinary least squares fit results for the level of **6** as indicated by the IPC data.

OLS Regression Results						
**Dep. Variable:**	Impurity	**R-squared:**	0.94			
**Model:**	OLS	**Adj. R-squared:**	0.918			
**Method:**	Least Squares	**F-statistic:**	43.02			
		**Prob (F-statistic):**	1.18E-06			
**Time:**	11:36:44	**Log-Likelihood:**	-19.237			
**No. Observations:**	16	**AIC:**	48.47			
**Df Residuals:**	11	**BIC:**	52.34			
**Df Model:**	4					
**Covariance Type:**	nonrobust					
	**coef**	**std err**	**t**	**P>|t|**	**[0.025**	**0.975]**
**Intercept**	13.15	0.243	54.161	0	12.616	13.684
**BBMO**	-0.5625	0.243	-2.317	0.041	-1.097	-0.028
**NaOH**	-1.5	0.243	-6.178	0	-2.034	-0.966
**Temp**	-1.5125	0.243	-6.23	0	-2.047	-0.978
**Solvent**	-2.3	0.243	-9.473	0	-2.834	-1.766
**Omnibus:**	2.112	**Durbin-Watson:**	2.365			
**Prob(Omnibus):**	0.348	**Jarque-Bera (JB):**	1.638			
**Skew:**	0.724	**Prob(JB):**	0.441			
**Kurtosis:**	2.399	**Cond. No.**	1			

Isolation of an Analytical Sample of Step 2 Impurity (**6**)

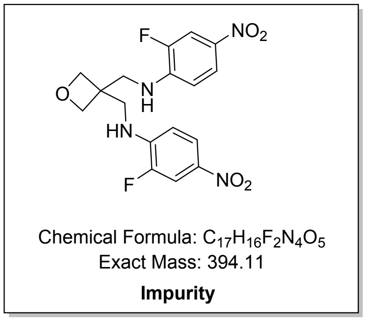

A sample of crude step 2 product (10 g, 90 area% of product **1** and 10 area% of impurity **6** by HPLC, Figure S5) was washed with methyl *tert*-butyl ether (MTBE, 20 mL x 3). The MTBE layer was then concentrated under reduced pressure to obtain sticky solid. The resulting solid was recrystallized from dichloromethane (2 V relative to recovered solid) at room temperature, filtered, washed with cold dichloromethane and dried under vacuum at room temperature to obtain impurity **6** in more than 95% HPLC purity. ^{1}H NMR (600 MHz, CD_3OD) δ 7.90-7.86 (m, 4H), 6.86-6.83 (t, J=9 Hz, 2H), 4.60 (s, 4H), 3.69 (s, 4H); ^{13}C NMR (151 MHz, DMSO-d_6) δ 149.2, 147.6, 143.9, 143.8, 134.8, 134.7, 122.4 (2C), 110.7, 110.5, 109.8, 109.8, 75.8 (2C), 45.4, 44.8 (2C); HRMS (ESI) m/z: calcd for $C_{17}H_{16}F_2N_4O_5Na$ $[M + Na]^+$, 417.0986; found, 417.0984.

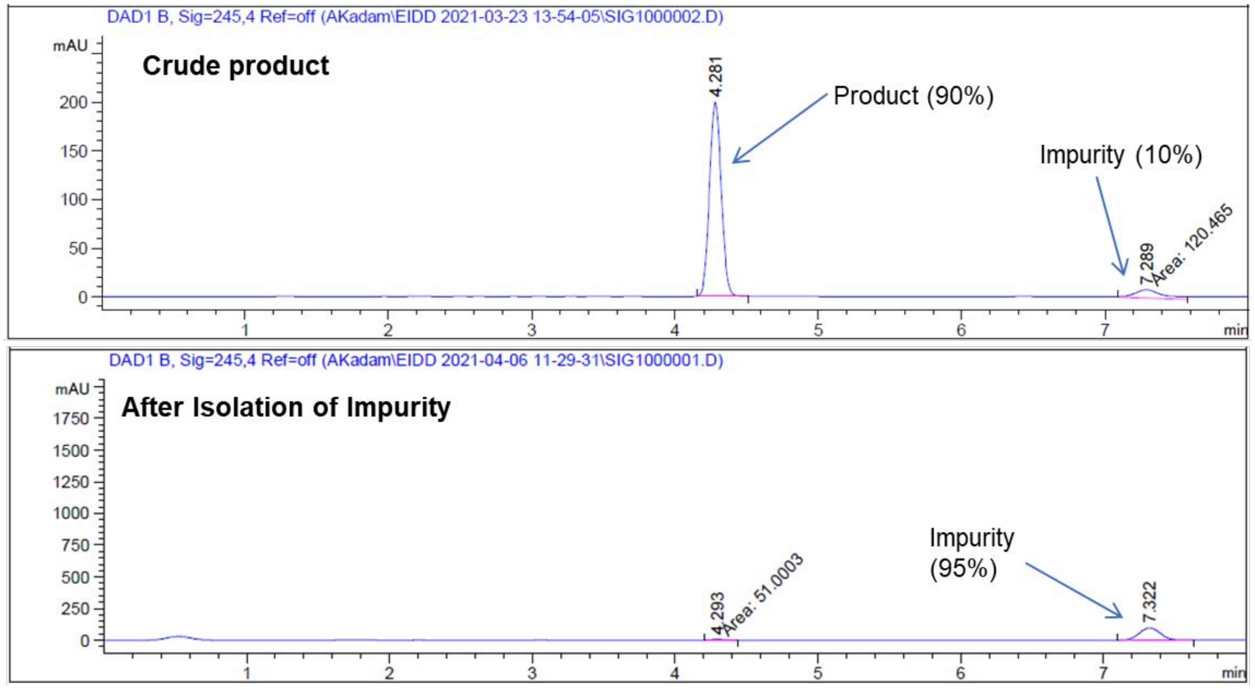

Figure S2: HPLC traces of crude product and impurity after isolation (for chromatography conditions, see below).

HPLC Data of Reaction Mixture and Product

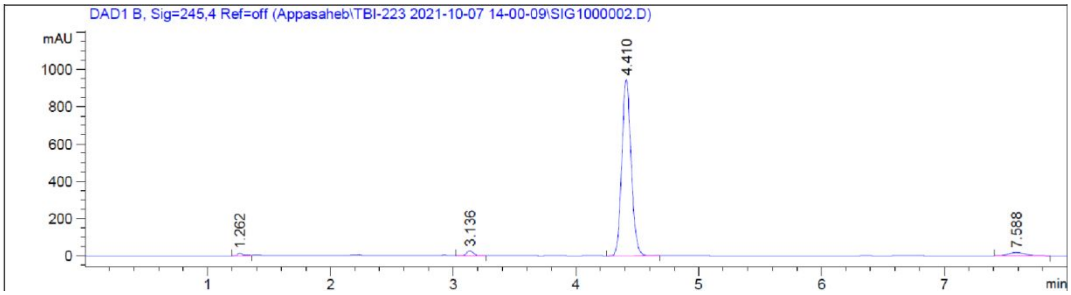

Peak #	RetTime [min]	Type	Width [min]	Area [mAU*s]	Height [mAU]	Area %	
1	1.262	BV	0.0441	45.51980	14.31805	0.8584	
2	3.136	VB	0.0570	104.59904	28.39412	1.9724	
3	4.410	VB	0.0817	4990.54199	944.15985	94.1064	Product
4	7.588	BB	0.1480	162.42340	17.07374	3.0628	Impurity

Figure S3: HPLC chromatogram of reaction mixture after completion (IPC; for chromatography conditions, see below).).

AK-TBI-071-135g

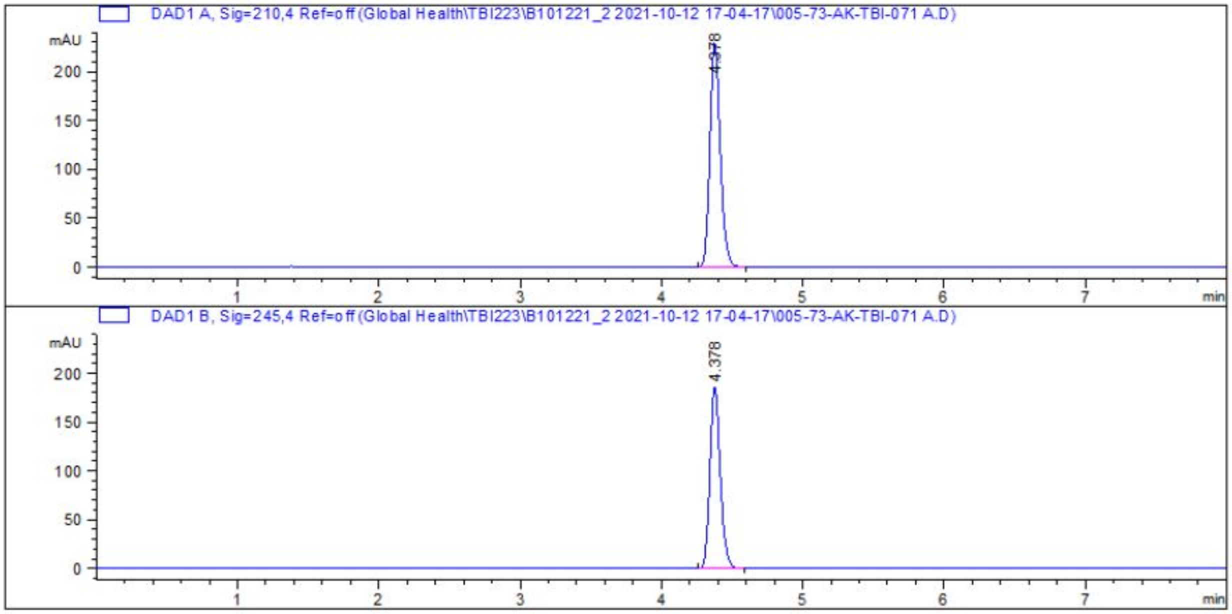

Figure S4: HPLC chromatograms of isolated product (100 area%; for chromatography conditions, see below).).

Photos of the Reaction and Isolated Product

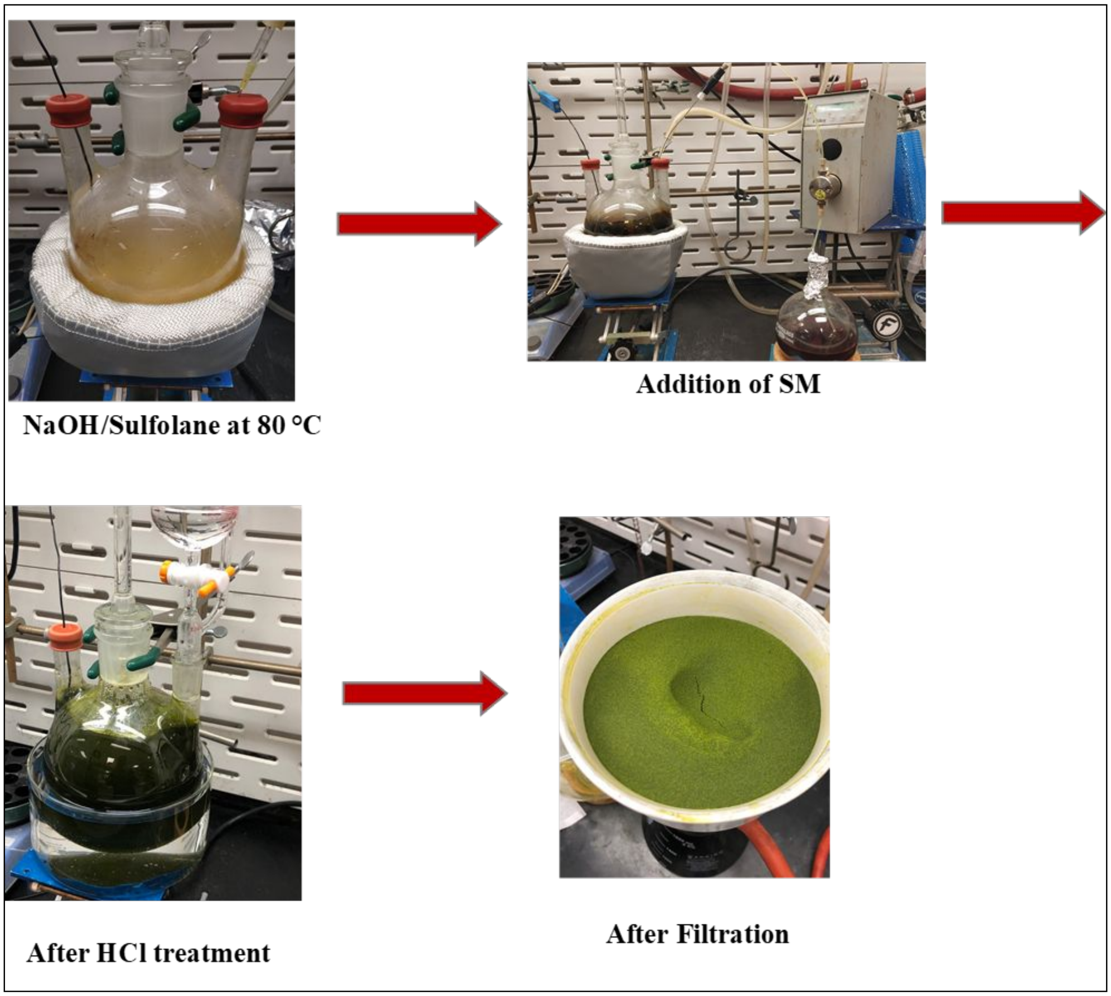

Figure S5: Photos taken at various stages of the reaction and isolation.

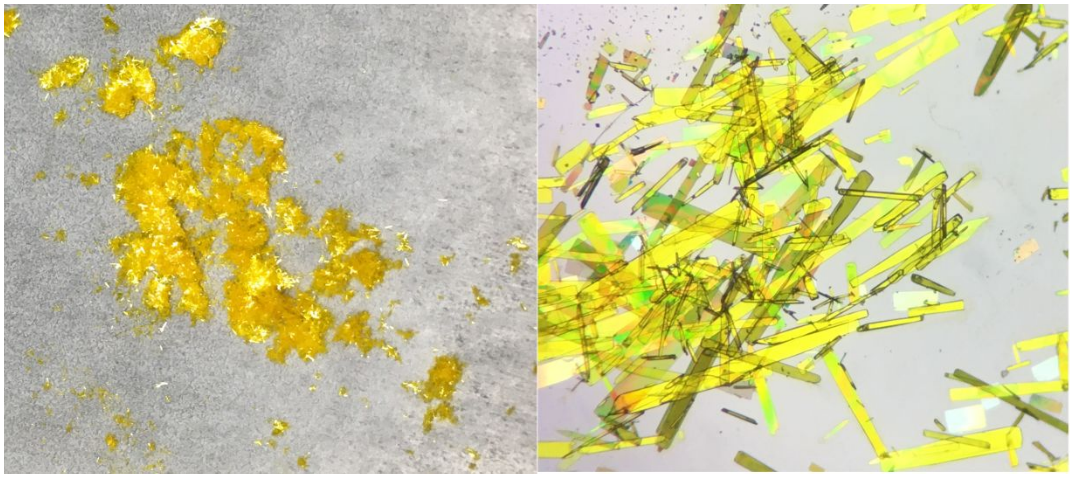

Figure S6: Samples of **1** crystallized from ethanol (right image taken under a polarizing microscope).

References

(1) Statsmodels - statistical models, hypothesis tests, and data exploration https://www.statsmodels.org/stable/index.html (accessed 2022 -11 -14).

NMR Spectra

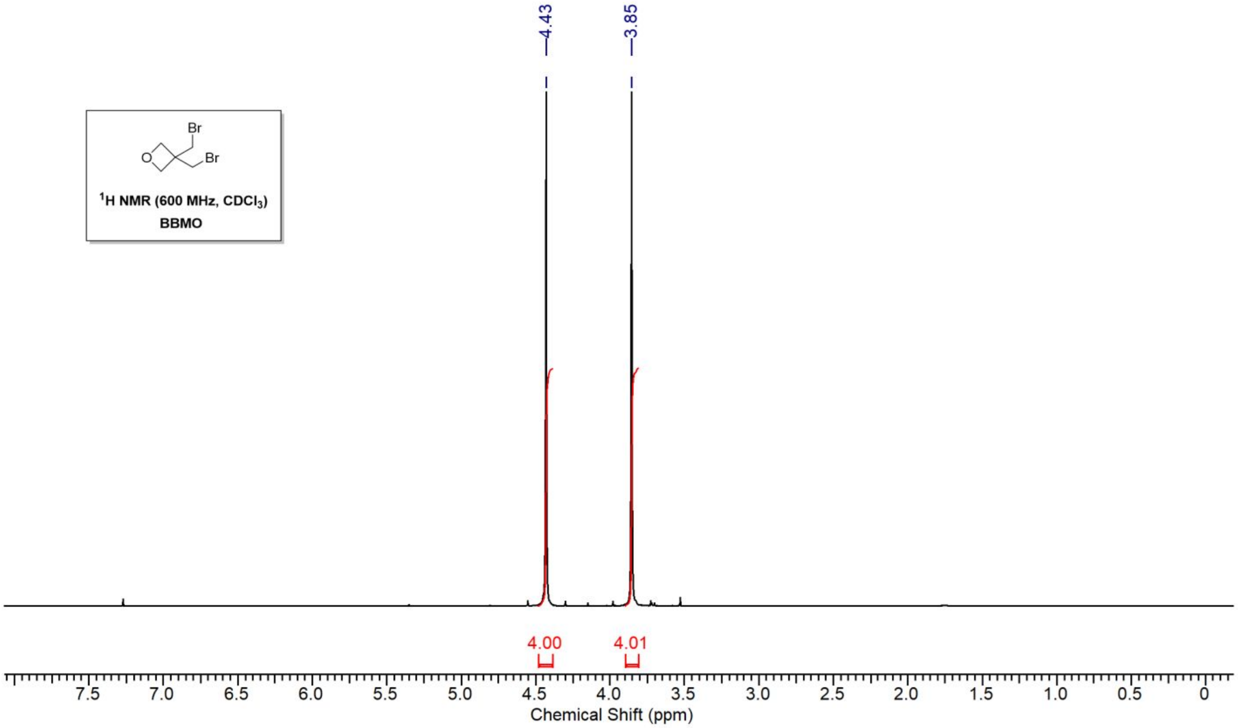

4.43
3.85
Br
O
Br
^{1}H NMR (600 MHz, CDCl$_3$)
BBMO
4.00
4.01
7.5
7.0
6.5
6.0
5.5
5.0
4.5
4.0
3.5
3.0
2.5
2.0
1.5
1.0
0.5
0
Chemical Shift (ppm)

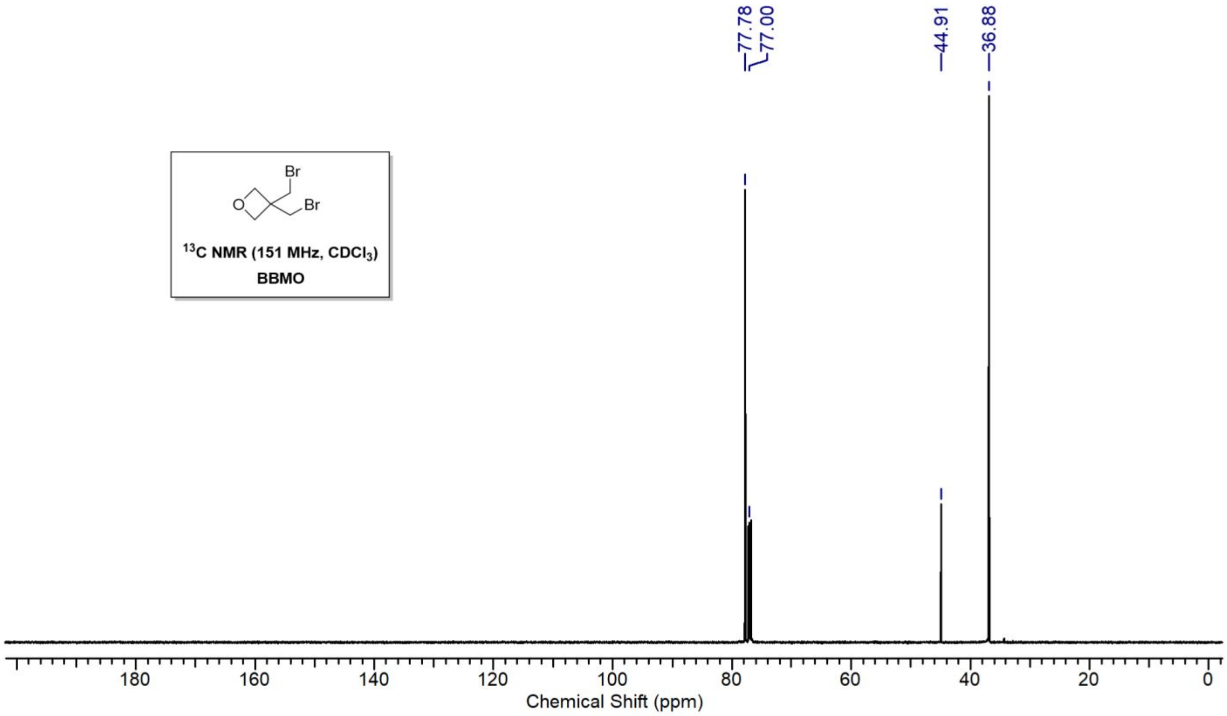

77.78
77.00
44.91
36.88
Br
O
Br
^{13}C NMR (151 MHz, CDCl$_3$)
BBMO
180
160
140
120
100
80
60
40
20
0
Chemical Shift (ppm)

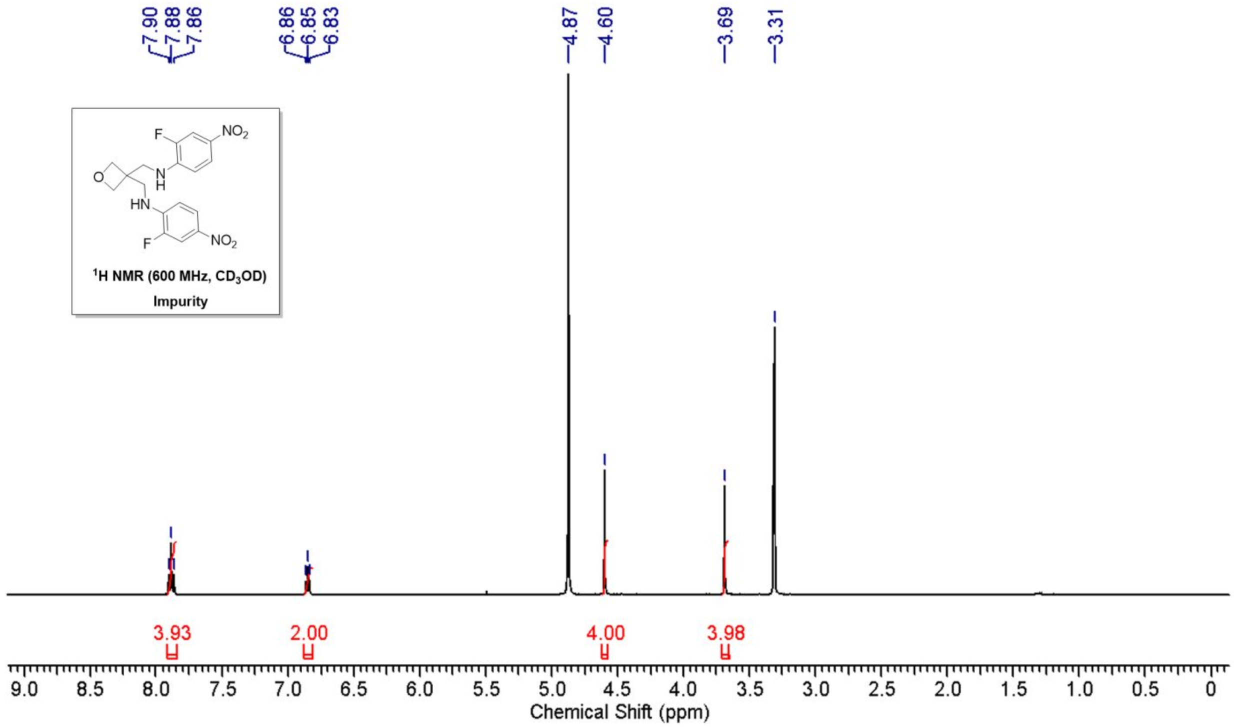
7.90
7.88
7.86
6.86
6.85
6.83
4.87
4.60
3.69
3.31
F
NO_2
O
N
H
HN
F
NO_2
^{1}H NMR (600 MHz, CD_3OD)
Impurity
3.93
2.00
4.00
3.98
9.0 8.5 8.0 7.5 7.0 6.5 6.0 5.5 5.0 4.5 4.0 3.5 3.0 2.5 2.0 1.5 1.0 0.5 0
Chemical Shift (ppm)

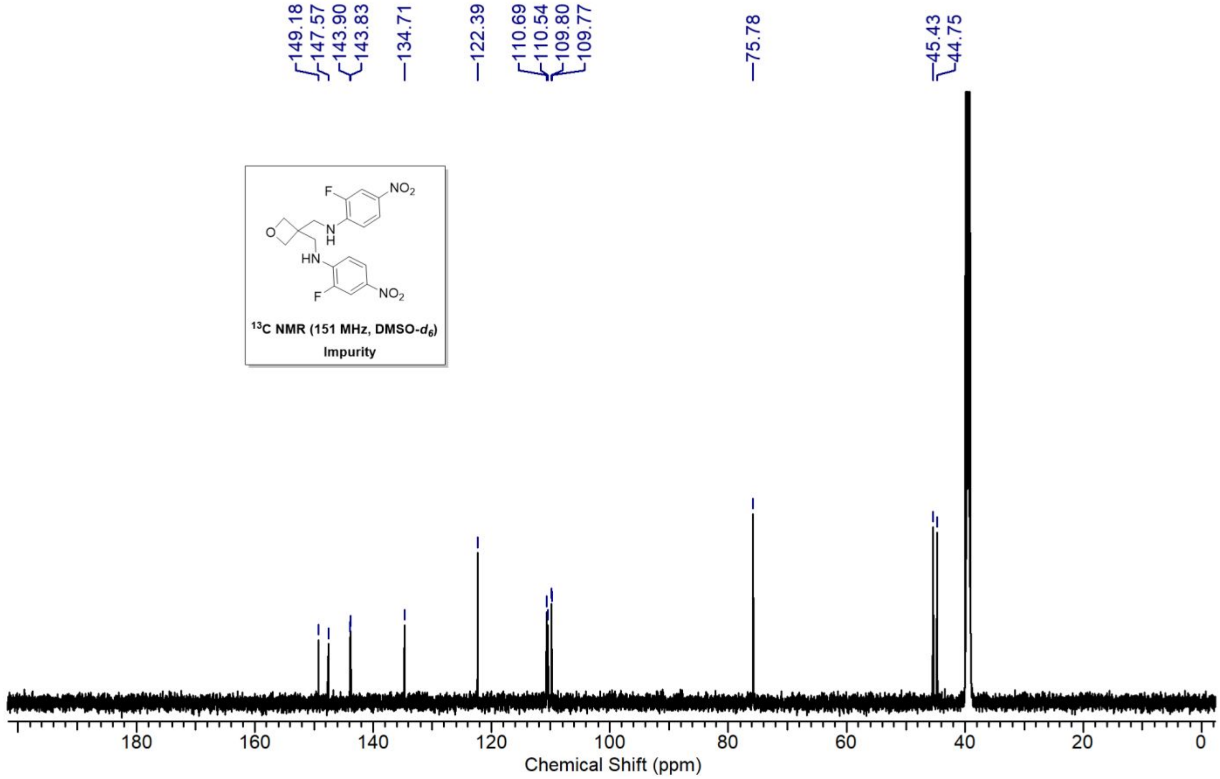
149.18
147.57
143.90
143.83
134.71
122.39
110.69
110.54
109.80
109.77
75.78
45.43
44.75
F
NO_2
O
N
H
HN
F
NO_2
^{13}C NMR (151 MHz, DMSO-d_6)
Impurity
180 160 140 120 100 80 60 40 20 0
Chemical Shift (ppm)

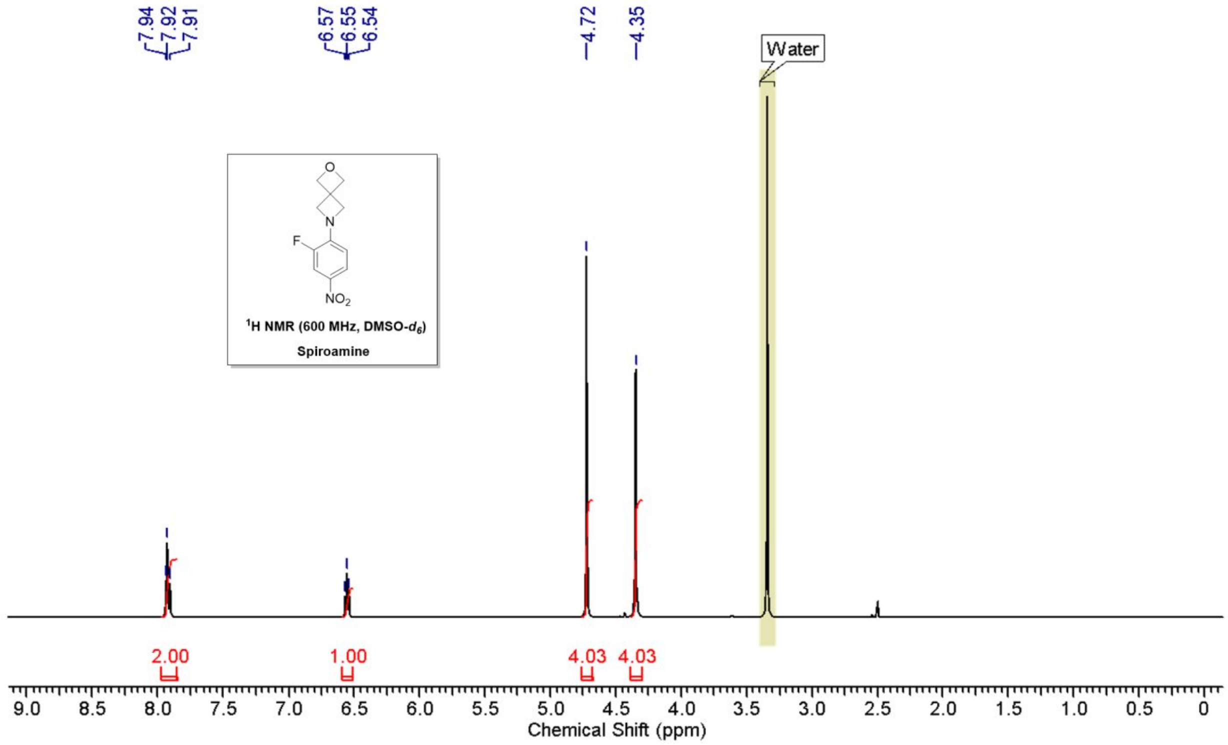
7.94
7.92
7.91
6.57
6.55
6.54
4.72
4.35
Water
O
N
F
NO2
1H NMR (600 MHz, DMSO-d6)
Spiroamine
2.00
1.00
4.03
4.03
Chemical Shift (ppm)

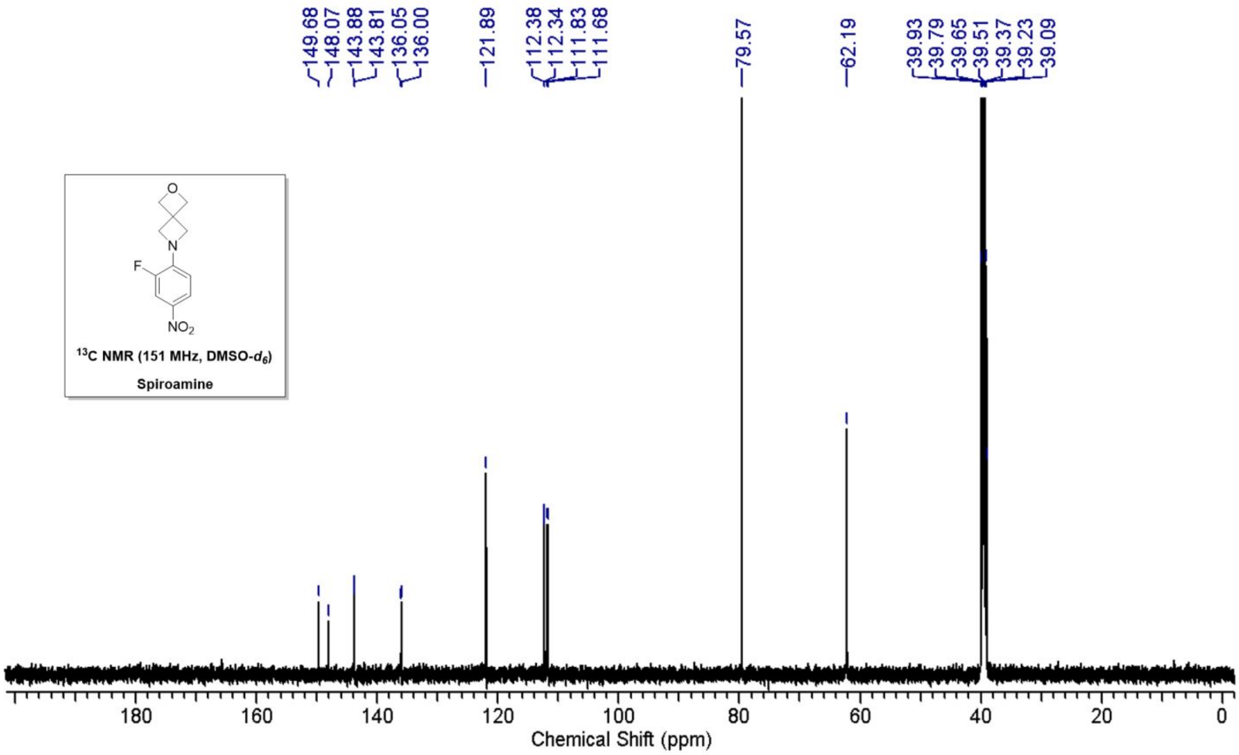
149.68
148.07
143.88
143.81
136.05
136.00
121.89
112.38
112.34
111.83
111.68
79.57
62.19
39.93
39.79
39.65
39.51
39.37
39.23
39.09
O
N
F
NO2
13C NMR (151 MHz, DMSO-d6)
Spiroamine
Chemical Shift (ppm)

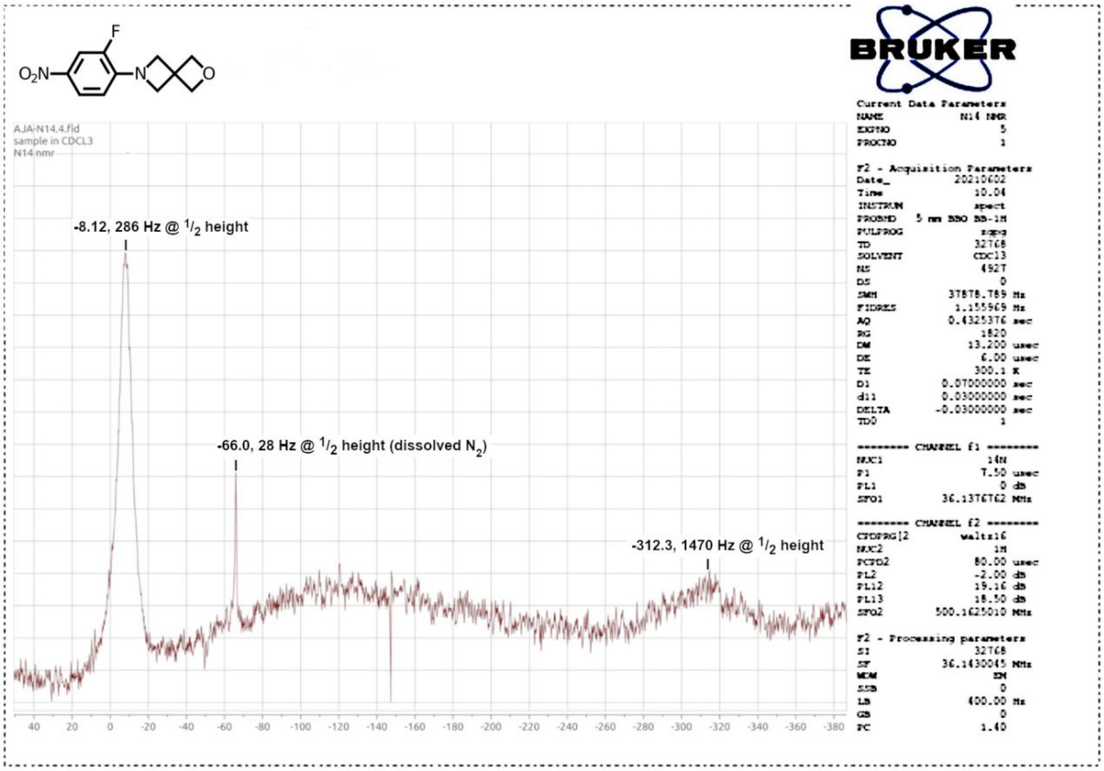
F
O2N
N
O
AJA-N14.4.fid
sample in CDCL3
N14 nmr
-8.12, 286 Hz @ 1/2 height
-66.0, 28 Hz @ 1/2 height (dissolved N2)
-312.3, 1470 Hz @ 1/2 height
40 20 0 -20 -40 -60 -80 -100 -120 -140 -160 -180 -200 -220 -240 -260 -280 -300 -320 -340 -360 -380
Current Data Parameters
NAME N14 NMR
EXPNO 5
PROCNO 1
F2 - Acquisition Parameters
Date_ 20210602
Time 10.04
INSTRUM spect
PROBHD 5 mm BBO BB-1H
PULPROG zgpg
TD 32768
SOLVENT CDCl3
NS 4927
DS 0
SWH 37878.789 Hz
FIDRES 1.155969 Hz
AQ 0.4325376 sec
RG 1820
DW 13.200 usec
DE 6.00 usec
TE 300.1 K
D1 0.07000000 sec
d11 0.03000000 sec
DELTA -0.03000000 sec
TD0 1
======== CHANNEL f1 ========
NUC1 14N
P1 7.50 usec
PL1 0 dB
SFO1 36.1376762 MHz
======== CHANNEL f2 ========
CPDPRG[2 waltz16
NUC2 1H
PCPD2 80.00 usec
PL2 -2.00 dB
PL12 19.16 dB
PL13 18.50 dB
SFO2 500.1625010 MHz
F2 - Processing parameters
SI 32768
SF 36.1430045 MHz
WDW EM
SSB 0
LB 400.00 Hz
GB 0
PC 1.40

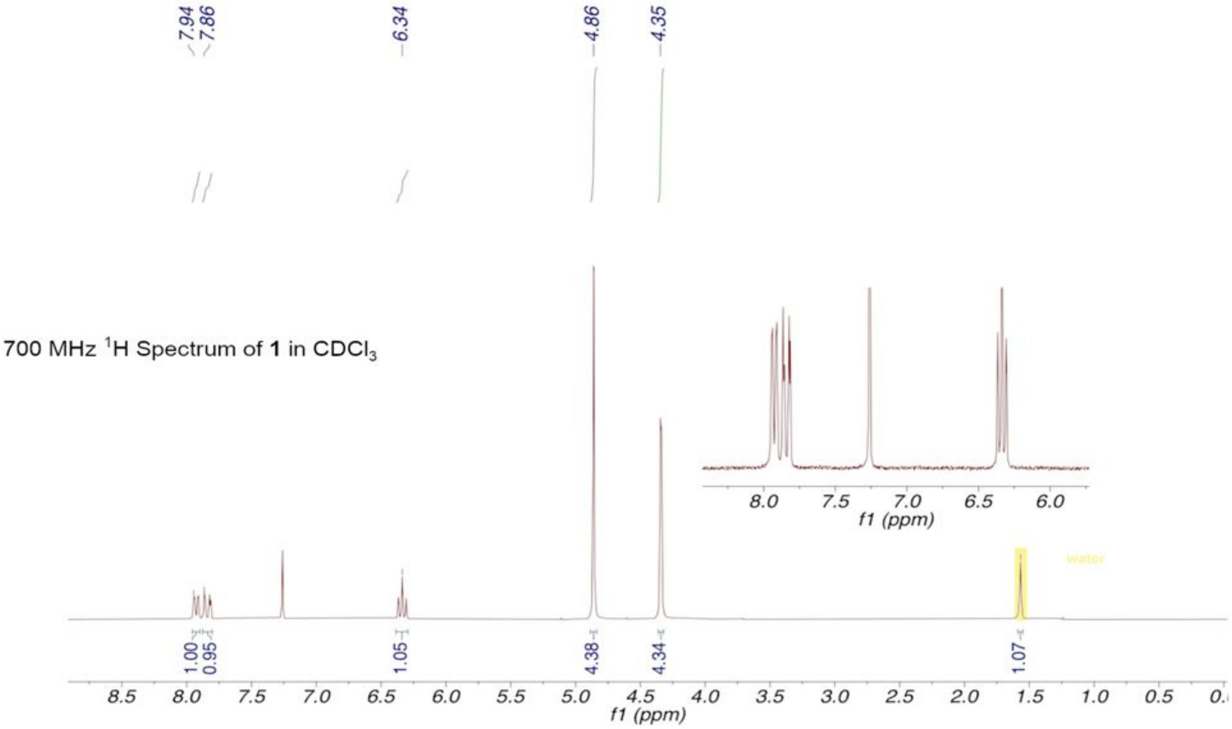
7.94
7.86
6.34
4.86
4.35
700 MHz 1H Spectrum of 1 in CDCl3
8.0 7.5 7.0 6.5 6.0
f1 (ppm)
water
1.00
0.95
1.05
4.38
4.34
1.07
8.5 8.0 7.5 7.0 6.5 6.0 5.5 5.0 4.5 4.0 3.5 3.0 2.5 2.0 1.5 1.0 0.5 0.
f1 (ppm)

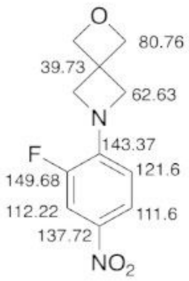

176 MHz ^{13}C Spectrum of **1** in $CDCl_3$

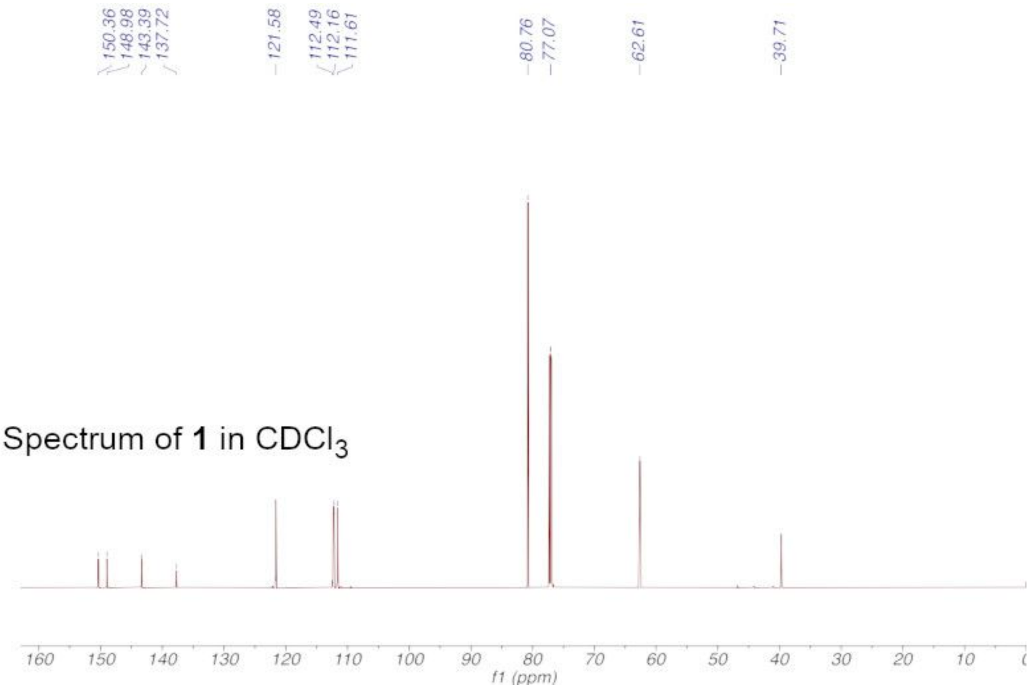

658.8 MHz ^{19}F Spectrum of **1** in $CDCl_3$ ($CFCl_3$ ref.)

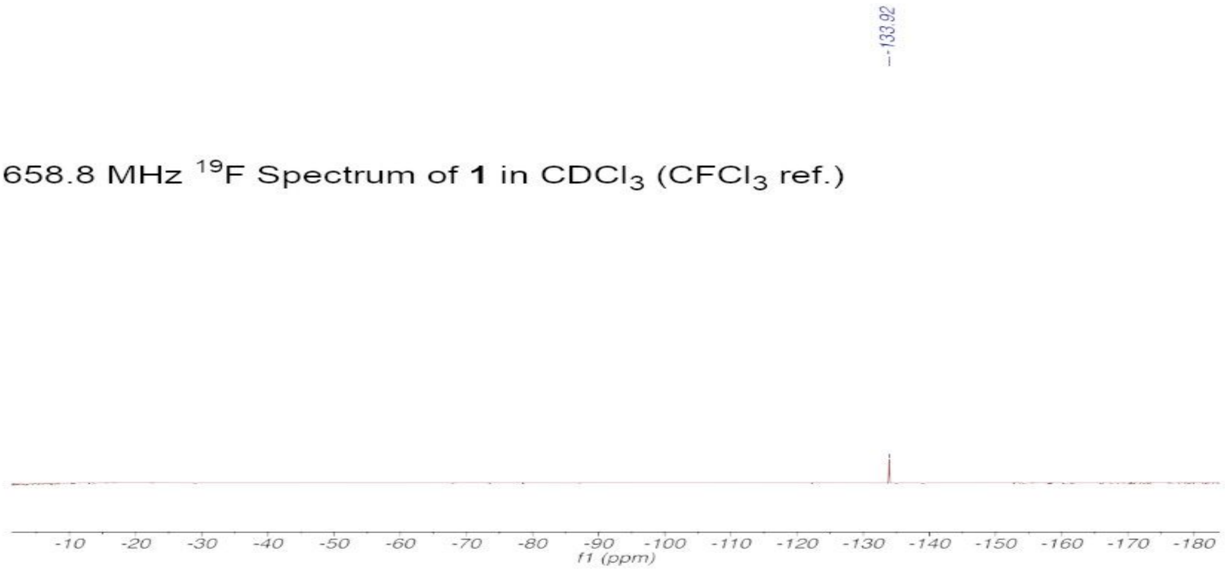

GCMS Method Report

Instrument: Agilent 6890 gas chromatograph with a 5977 mass selective (MSD) detector

GC-MSD Method Report

medicines for all institute

Project: TBI-223
Purpose: To separate TBI-223 starting material and intermediates

Date: August 16, 2021
Method ID: DWC_Iso200Thick.M

Structures & IDs:

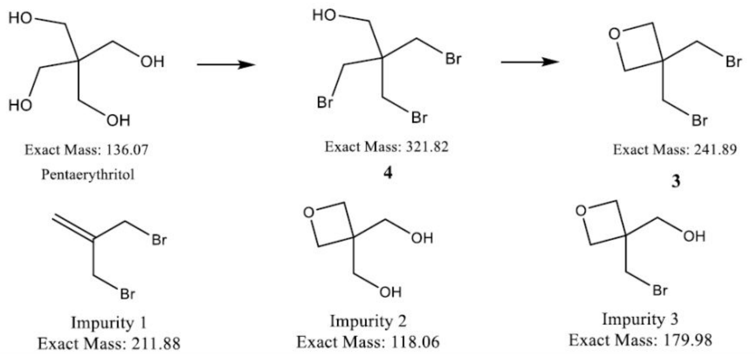

Conditions:

Column: HP-5ms 30 m x 250 µm x 5 µm

Inlet Pressure: 16.5 psi
Split Ratio: 50:1
Split Flow: 70 mL/min
Column flow: 0.8 mL/min
Injection Temp: 225 °C
Injection volume: 1 µL
Solvent Delay: 1.2 min
Runtime: 25 min

Temperature Program:

Time (min)	Temp (°C)	Ramp (°C/min)	Hold (min)
0	200	-	25

MS Parameters:

Transfer Line Temp (°C)	250
Source Temp (°C)	230
Quad Temp (°C)	150
Electron Energy (eV)	70

Sample preparation: Samples are prepared in acetonitrile with the exception of pentaerythritol which should be prepared in methanol.

Retention Times

**Compound**	***m/z***	**Time (min)**
Impurity 1	211	2.4
Impurity 2	87 (fragment), 70 (fragment)	3.5
Impurity 3	150 (fragment), 71 (fragment)	4.1
**3**	211 (fragment), 133 (fragment)	4.7
Pentaerythritol	70 (fragment)	6.4
**4**	212 (fragment), 133 (fragment)	13.1

Notes:

Page **1** of **3**

Representative Chromatogram(s) (attach additional chromatograms and spectra as needed)

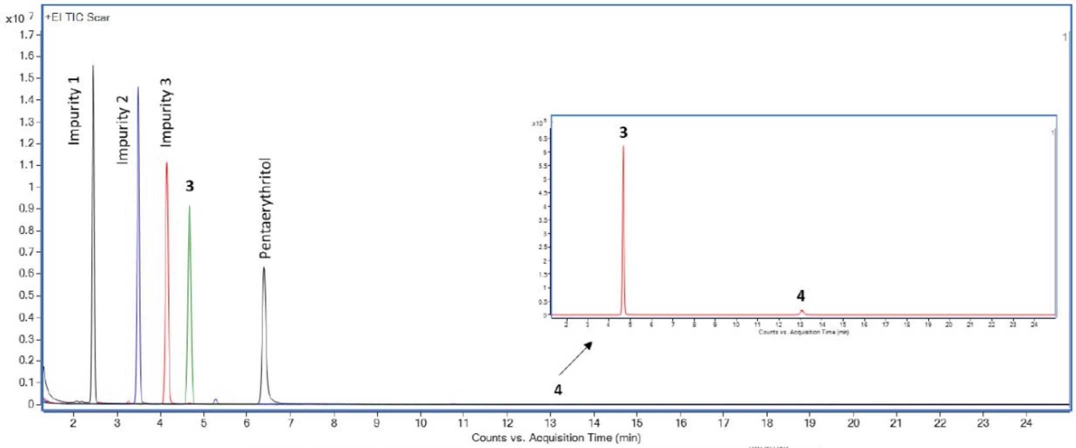

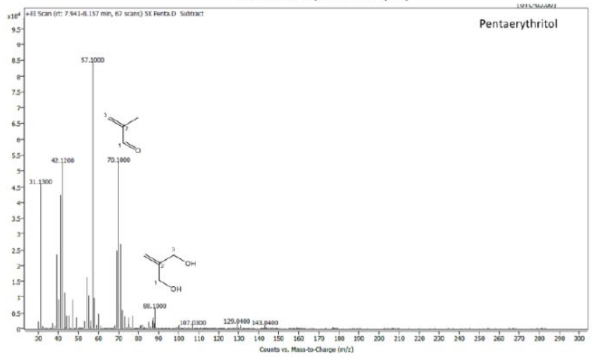

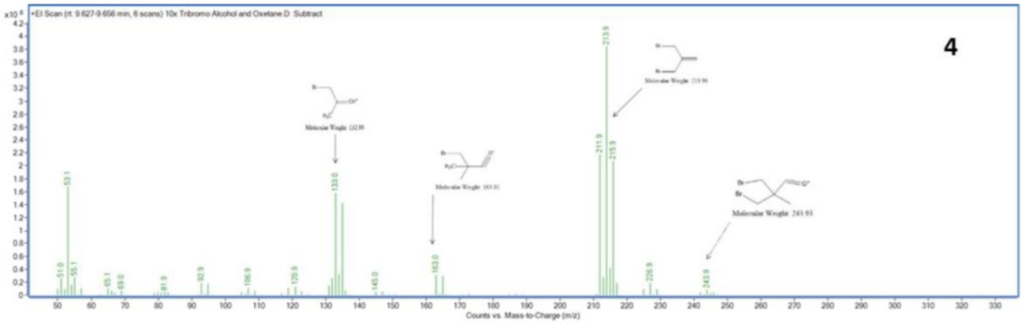

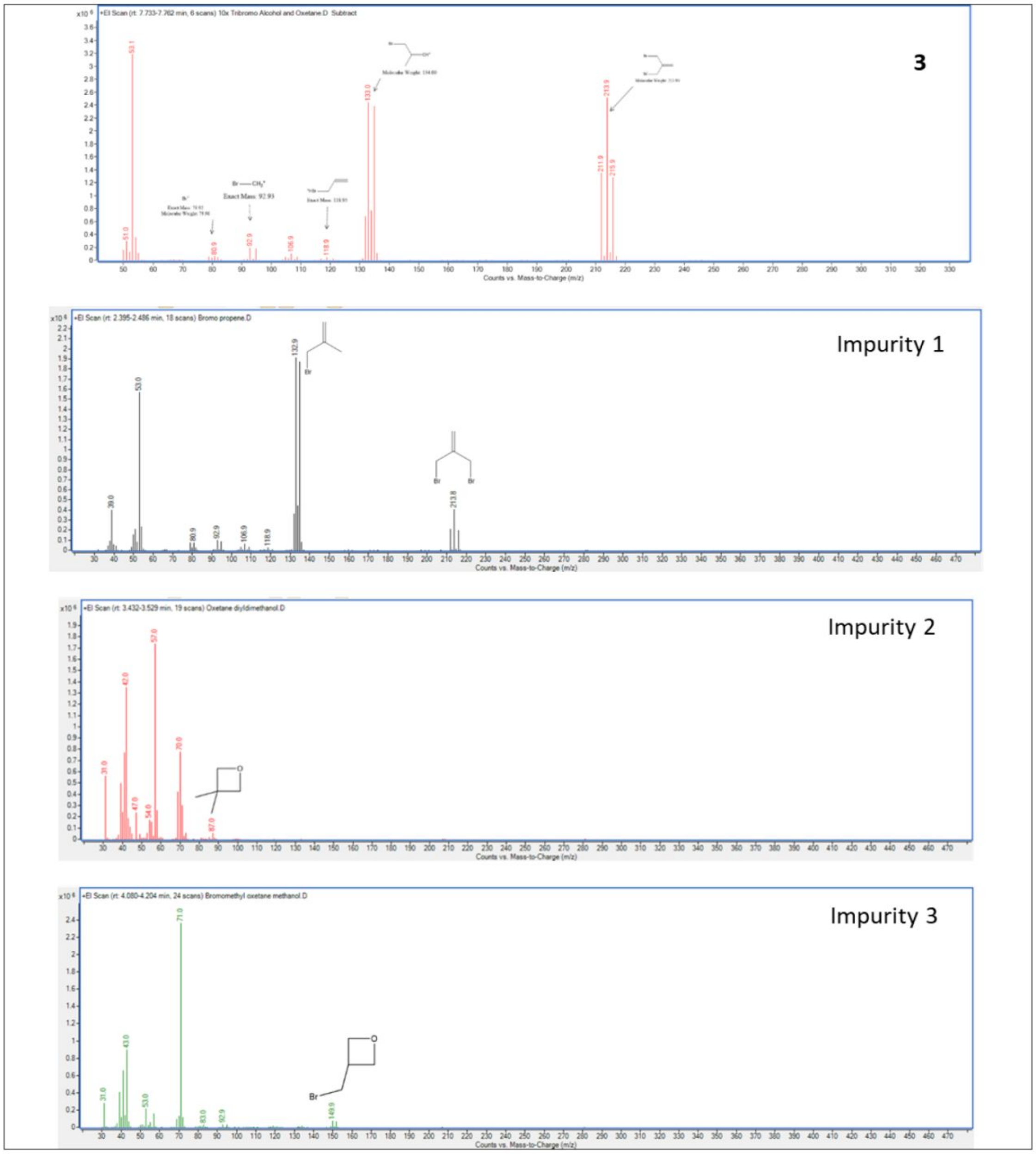
3
Impurity 1
Impurity 2
Impurity 3
Counts vs. Mass-to-Charge (m/z)

HPLC-UV Method Report

Instrument: Agilent 1100 LC unit with a diode array detector

HPLC-UV Method Report

medicines for all institute

Project: TBI-223
Purpose: To separate compounds 1 and 2

Date: January 25, 2021
Method ID: TBI_Isocratic 50%ACN

Structures & IDs:

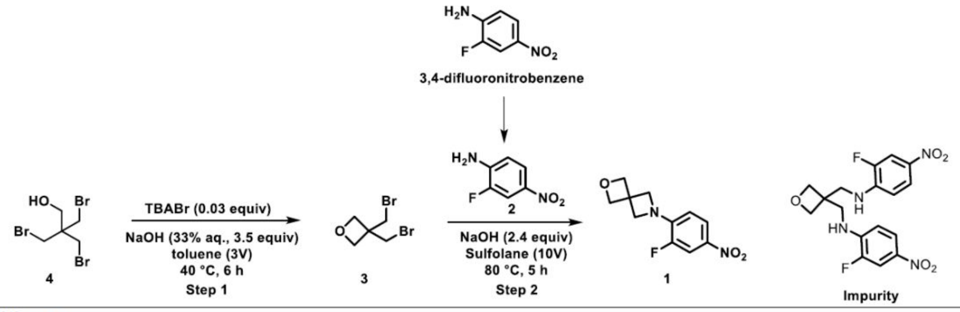

Conditions:

Column: Agilent Zorbax Eclipse XDB-C18 (4.6 x 250 mm; 5 µm)

Mobile Phase A: 0.1% Phosphoric Acid in Water (1 mL HPLC grade H_3PO_4 in 1000 mL HPLC grade water)

Mobile Phase B: Acetonitrile

Injection volume: 1 µL   Column temp: 30 °C   Flow rate: 1.5 mL/min

Detector wavelength(s): 245 nm (primary), 210 nm (monitoring)

LC Gradient Table:

Time (min)	%A	%B
0	50	50
8	50	50

Post-run equilibration: 0 min

Sample preparation: Samples are prepared at ~1 mg/mL in acetonitrile.

Retention Times

Compound	Time (min)	Relative RF (mg/mL)*	Relative RF (M)*
**2**	3.1	0.71	1.1
**1**	4.4	1.0	1.0
3,4-Difluoronitrobenzene	5.5	0.81	1.2
Impurity	7.3	-	-

Notes:

3 and **4** are not detectable by UV and should be analyzed by GC-MS.

$$*Relative\ RF = \frac{\left(^{Analyte\ 2\ Conc.}/_{Analyte\ 2\ Peak\ Area}\right)}{\left(^{Spiroamine\ Conc.}/_{Spiroamine\ Peak\ Area}\right)}$$

Representative Chromatogram(s) (attach additional chromatograms and spectra as needed)

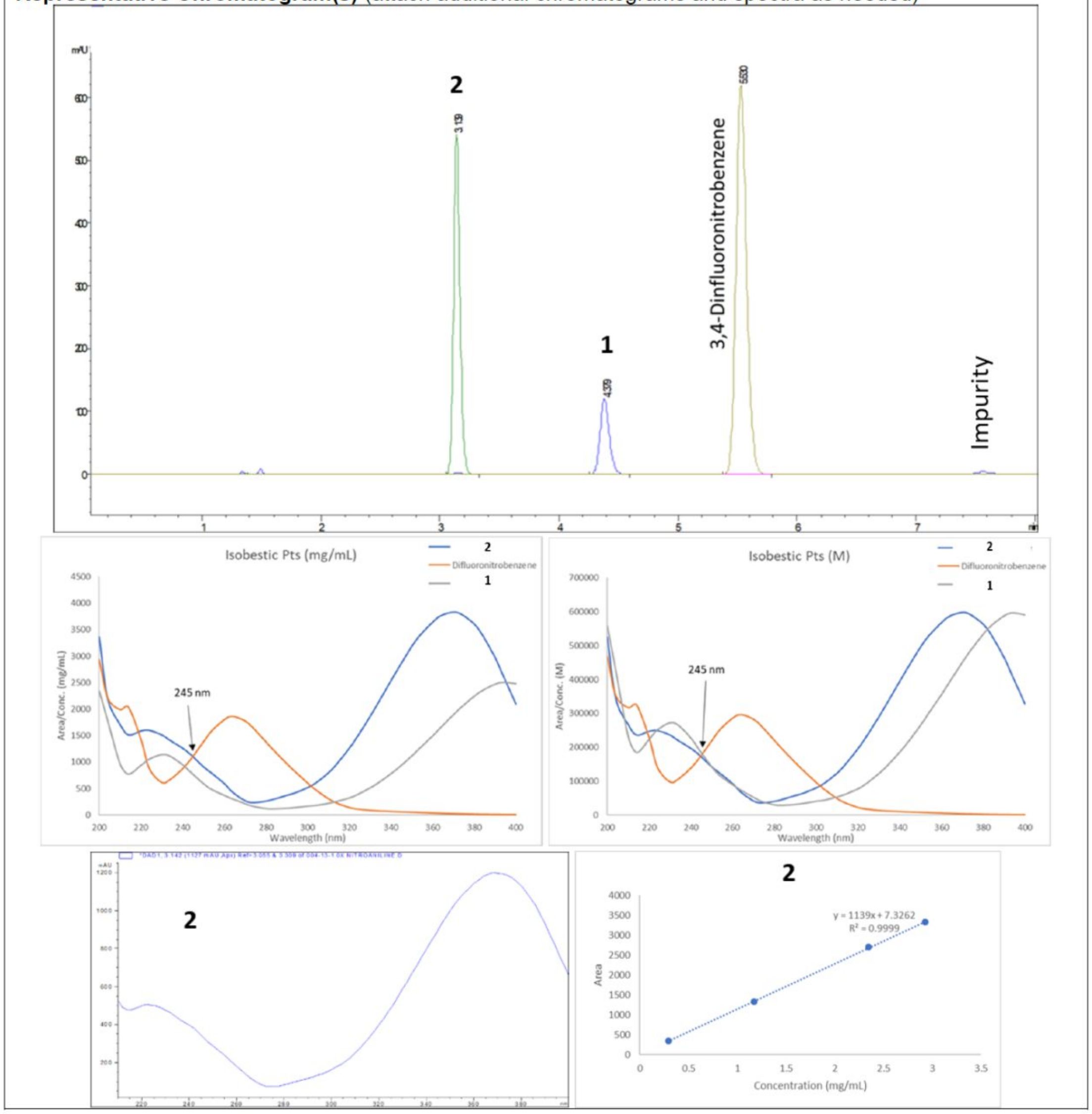

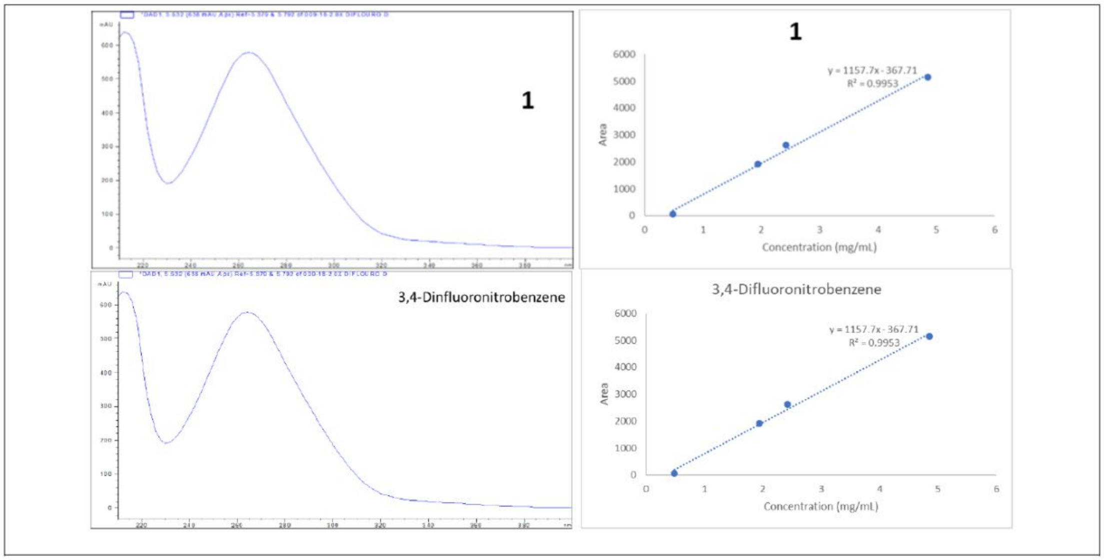
1
1
y = 1157.7x - 367.71
R² = 0.9953
Area
Concentration (mg/mL)
3,4-Dinfluoronitrobenzene
3,4-Difluoronitrobenzene
y = 1157.7x - 367.71
R² = 0.9953
Area
Concentration (mg/mL)

X-Ray Crystallographic Data and Analysis

Crystal Data and Experimental on **1**.

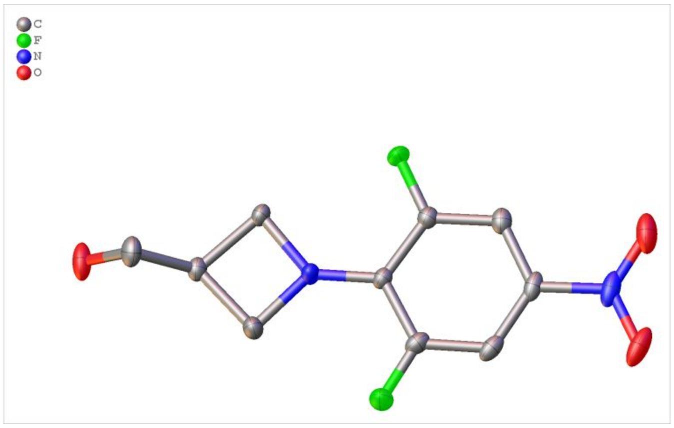

*ORTEP of **1***. Only unique and disordered positions with hydrogens omitted, (all data) and R_1 was 0.0412 (I≥2 σ(I)).

Experimental. Single clear yellow prism-shaped crystals of **AJA03A-Qu** were used as supplied. A suitable crystal with dimensions 0.38 × 0.24 × 0.12 mm³ was selected and mounted on a XtaLAB Synergy R, DW system, HyPix diffractometer. The crystal was kept at a steady T = 100.01(10) K during data collection. The structure was solved with the **ShelXT** 2014/5 (Sheldrick, 2014) solution program using dual methods and by using **Olex2** 1.3-alpha (Dolomanov et al., 2009) as the graphical interface. The model was refined with **ShelXL** 2018/3 (Sheldrick, 2015) using full matrix least squares minimisation on $\boldsymbol{F}^2$.

Crystal Data. $C_{11}H_{11}FN_2O_3$, M_r = 238.22, monoclinic, $P2_1/m$ (No. 11), a = 8.2926(5) Å, b = 6.6406(4) Å, c = 10.1213(6) Å, β = 112.811(7)°, α = γ = 90°, V = 513.77(6) Å³, T = 100.01(10) K, Z = 2, Z' = 0.5, μ(Mo K_α) = 0.125, 8766 reflections measured, 1775 unique (R_{int} = 0.0201) which were used in all calculations. The final wR_2 was 0.1189 (all data) and R_1 was 0.0412 (I≥2 σ(I)).

**Compound**	AJA03A-Qu (**1**)
Formula	$C_{11}H_{11}FN_2O_3$
$D_{calc.}$/ g cm^{-3}	1.540
μ/mm^{-1}	0.125
Formula Weight	238.22
Colour	clear yellow
Shape	prism-shaped
Size/mm³	0.38×0.24×0.12
T/K	100.01(10)
Crystal System	monoclinic
Space Group	$P2_1/m$
a/Å	8.2926(5)
b/Å	6.6406(4)
c/Å	10.1213(6)
α/°	90
β/°	112.811(7)
γ/°	90
V/Å³	513.77(6)
Z	2
Z'	0.5
Wavelength/Å	0.71073
Radiation type	Mo K_α
Θ_{min}/°	2.665
Θ_{max}/°	32.418
Measured Refl's.	8766
Indep't Refl's	1775
Refl's I≥2 σ(I)	1543
R_{int}	0.0201
Parameters	107
Restraints	0

Model. The raw data and refinement model were of good quality and excellent fit. The molecule sits on a crystallographic mirror plane that includes the azetidine and phenyl rings, the nitro-group, the fluorine atom, and the terminal ether oxygen of the oxetane ring. The orientation of the fluorophenyl moiety was modeled with disorder between two positions. One orientation (36%) placed the fluorine in the vicinity of a nitro-oxygen

(rinter-atomic = 2.62 Å) while the second orientation (64%) placed the fluorine into a small void adjacent the oxetane ring of a neighboring molecule (see Figure 2X). No evidence was found for a super-lattice that might eliminate disorder in the model.

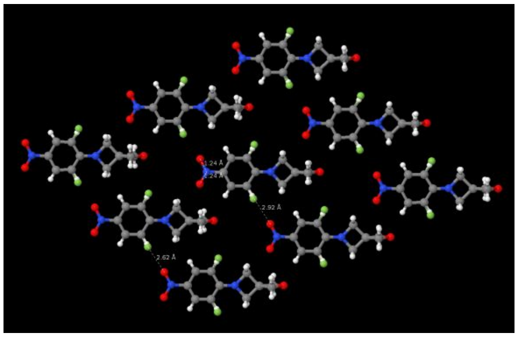

Figure *2X*: Planar packing diagram showing F-disorder.

Structure Quality Indicators

**Reflections:**	d min (Mo) 2θ=64.8° 0.66	I/σ(I) 57.8	Rint 2.01%	CAP 59.9° 90% to 64.8° 99.9
**Refinement:**	Shift 0.000	Max Peak 0.4	Min Peak -0.3	GooF 1.048

A clear yellow prism-shaped-shaped crystal with dimensions 0.38 × 0.24 × 0.12 mm^3 was mounted. Data were collected using a XtaLAB Synergy R, DW system, HyPix diffractometer operating at T = 100.01(10) K.

Data were measured using ω scans using Mo K_α radiation. The diffraction pattern was indexed and the total number of runs and images was based on the strategy calculation from the program **CrysAlisPro** (Rigaku, V1.171.41.102a, 2021). The maximum resolution that was achieved was Θ = 32.418° (0.66 Å).

The unit cell was refined using **CrysAlisPro** (Rigaku, V1.171.41.102a, 2021) on 5618 reflections, 64% of the observed reflections.

Data reduction, scaling and absorption corrections were performed using **CrysAlisPro** (Rigaku, V1.171.41.102a, 2021). The final completeness is 99.90 % out to 32.418° in Θ. A gaussian absorption correction was performed using CrysAlisPro 1.171.41.102a (Rigaku Oxford Diffraction, 2021)Numerical absorption correction based on gaussian integration overa multifaceted crystal modelEmpirical absorption correction using spherical harmonics, implemented in SCALE3 ABSPACK scaling algorithm.. The absorption coefficient μ of this material is 0.125 mm^{-1} at this wavelength (λ = 0.71073Å) and the minimum and maximum transmissions are 0.790 and 1.000.

The structure was solved and the space group $P2_1/m$ (# 11) determined by the ShelXT 2014/5 (Sheldrick, 2014) structure solution program using using dual methods and refined by full matrix least squares minimisation on $\boldsymbol{F^2}$ using version 2018/3 of ShelXL 2018/3 (Sheldrick, 2015). All non-hydrogen atoms were refined anisotropically. Hydrogen atom positions were calculated geometrically and refined using the riding model. Hydrogen atom positions were calculated geometrically and refined using the riding model.

_refine_special_details: The molecule was found to be disordered by a 180 degree rotation around the C-N bond. It was conveniently modeled as F atom 2-component positional disorder. No restraints or constraints were applied.

_exptl_absorpt_process_details: CrysAlisPro 1.171.41.102a (Rigaku Oxford Diffraction, 2021)Numerical absorption correction based on gaussian integration overa multifaceted crystal modelEmpirical absorption correction using spherical harmonics,implemented in SCALE3 ABSPACK scaling algorithm.

The value of Z' is 0.5. This means that only half of the formula unit is present in the asymmetric unit, with the other half consisting of symmetry equivalent atoms.

Crystallographic Citations

CrysAlisPro (Rigaku, V1.171.41.102a, 2021)

CrysAlisPro (ROD), Rigaku Oxford Diffraction, Poland.

Dolomanov, O.V., Bourhis, L.J., Gildea, R.J, Howard, J.A.K. & Puschmann, H. (2009), J. Appl. Cryst. 42, 339-341.Sheldrick, G.M. (2015). Acta Cryst. A71, 3-8.Sheldrick, G.M. (2015). Acta Cryst. C71, 3-8.

O.V. Dolomanov and L.J. Bourhis and R.J. Gildea and J.A.K. Howard and H. Puschmann, Olex2: A complete structure solution, refinement and analysis program, *J. Appl. Cryst.*, (2009), **42**, 339-341.

Sheldrick, G.M., Crystal structure refinement with ShelXL, *Acta Cryst.*, (2015), **C71**, 3-8.

Sheldrick, G.M., ShelXT-Integrated space-group and crystal-structure determination, *Acta Cryst.*, (2015), A71, 3-8.
